# Epigenomics of conventional type-I dendritic cells depicted preferential control of TLR9 versus TLR3 response by NCoR1 through differential IRF3 activation

**DOI:** 10.1007/s00018-022-04424-w

**Published:** 2022-07-18

**Authors:** Gyan Prakash Mishra, Atimukta Jha, Abdul Ahad, Kaushik Sen, Aishwarya Sen, Sreeparna Podder, Subhasish Prusty, Viplov Kumar Biswas, Bhawna Gupta, Sunil Kumar Raghav

**Affiliations:** 1 Immuno-Genomics and Systems Biology Laboratory, Institute of Life Sciences (ILS), Bhubaneswar, Odisha 751023 India; 2 School of Biotechnology, Kalinga Institute of Industrial Technology (KIIT), Bhubaneswar, Odisha 751024 India; 3 Manipal Academy of Higher Education, Manipal, Karnataka 576104 India; 4 Regional Centre for Biotechnology, Faridabad, Haryana 121001 India

**Keywords:** Dendritic cells, TLR activation, NCoR1, Immune response, Enhancer activity, Multi-omics data analysis (RNA-seq and ChIP-seq)

## Abstract

Tight control of gene regulation in dendritic cells (DCs) is important to mount pathogen specific immune responses. Apart from transcription factor binding, dynamic regulation of enhancer activity through global transcriptional repressors like Nuclear Receptor Co-repressor 1 (NCoR1) plays a major role in fine-tuning of DC responses. However, how NCoR1 regulates enhancer activity and gene expression in individual or multiple Toll-like receptor (TLR) activation in DCs is largely unknown. In this study, we did a comprehensive epigenomic analysis of murine conventional type-I DCs (cDC1) across different TLR ligation conditions. We profiled gene expression changes along with H3K27ac active enhancers and NCoR1 binding in the TLR9, TLR3 and combined TLR9 + TLR3 activated cDC1. We observed spatio-temporal activity of TLR9 and TLR3 specific enhancers regulating signal specific target genes. Interestingly, we found that NCoR1 differentially controls the TLR9 and TLR3-specific responses. NCoR1 depletion specifically enhanced TLR9 responses as evident from increased enhancer activity as well as TLR9-specific gene expression, whereas TLR3-mediated antiviral response genes were negatively regulated. We validated that NCoR1 KD cDC1 showed significantly decreased TLR3 specific antiviral responses through decreased IRF3 activation. In addition, decreased IRF3 binding was observed at selected ISGs leading to their decreased expression upon NCoR1 depletion. Consequently, the NCoR1 depleted cDC1 showed reduced Sendai Virus (SeV) clearance and cytotoxic potential of CD8^+^ T cells upon TLR3 activation. NCoR1 directly controls the majority of these TLR specific enhancer activity and the gene expression. Overall, for the first time, we revealed NCoR1 mediates transcriptional control towards TLR9 as compared to TLR3 in cDC1.

**Graphical abstract:**

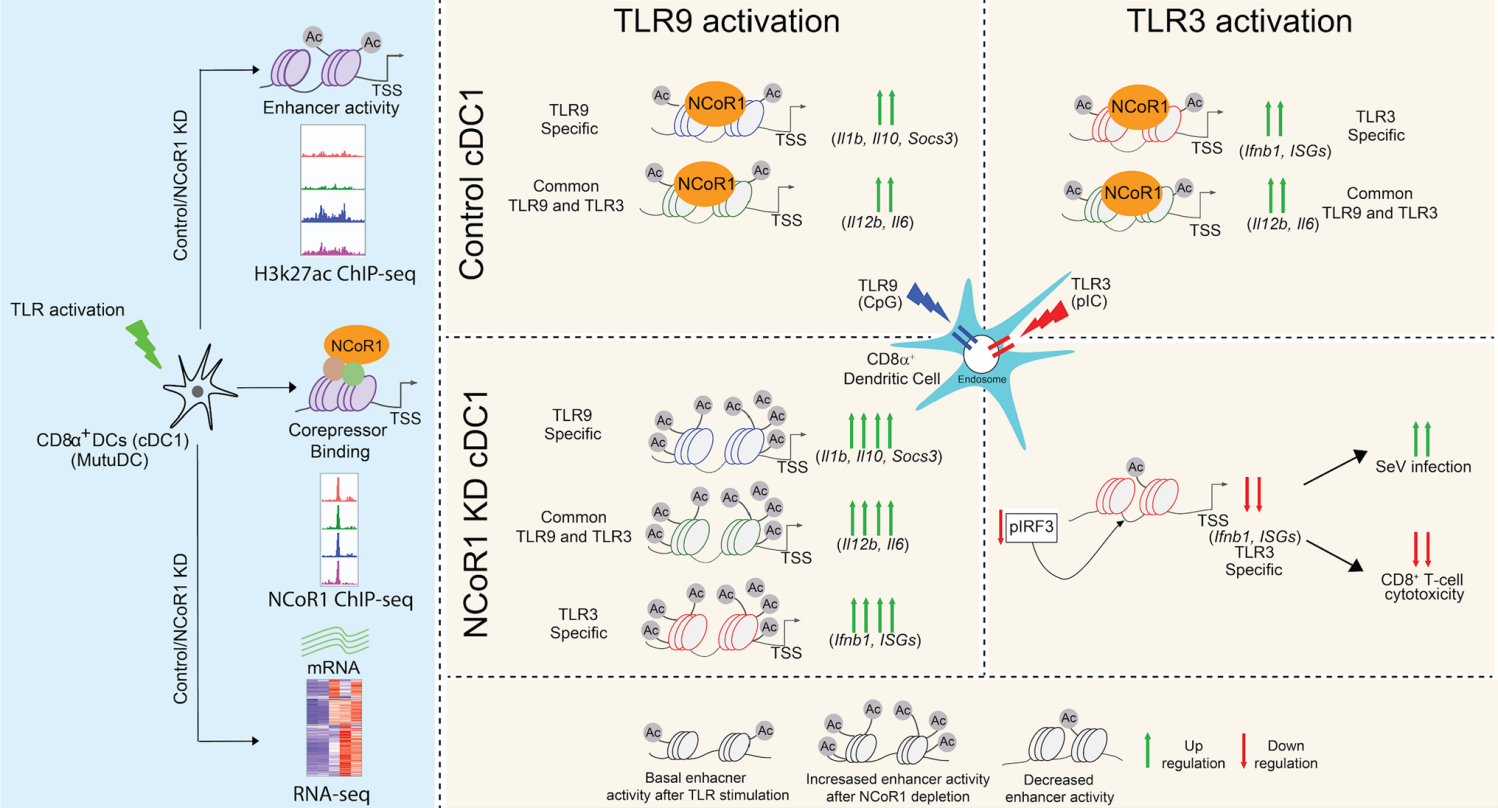

**Supplementary Information:**

The online version contains supplementary material available at 10.1007/s00018-022-04424-w.

## Introduction

Type 1 classical or conventional CD8α^+^ Dendritic cells (cDC1) are important sentinel of adaptive immunity. They are known to control the balance between multiple immune responses such as inflammatory/anti-inflammatory and antiviral against bacteria and viruses, respectively [1]. cDC1 can recognize multiple pathogen associated molecular patterns (PAMPs) through various toll-like receptors (TLRs) present either on cell surfaces or inside the cells on endoplasmic reticulum or endosomes [2–5]. Apart from PAMPs recognition, cDC1 can also encounter host response factors such as IFNγ secreted from T-cells that binds to Type-II IFN receptor (IFNGR) and enhances MHC I/MHC II expression, activation and maturation of DCs [6–9]. Downstream signaling under TLRs activates transcription factors such as nuclear factor kappa-light-chain-enhancer of activated B cells (NF-kB) or Interferon regulatory factors (IRFs) either in myeloid differentiation primary response 88 (MyD88) or toll/IL-1 receptor (TIR) domain-containing adaptor (TRIF)-dependent manner [10–12]. All TLRs except TLR3 activate downstream signaling through MyD88 adaptor protein followed by phosphorylation of TNF receptor-associated factor (TRAF) family of proteins, mainly TRAF6 that leads to activation of NF-kB [13]. On the other hand, TLR3 signals through TRIF adaptor proteins and activates IRF3 and NF-kB [14]. Though these signal related transcription factors (SRTFs) are specific to some TLRs or common for one or the other TLRs, the spatio-temporal changes in gene expression is dynamically regulated through enhancer activity that further defines the cell state and function [15–17]. Transcription factor binding to the accessible chromatin regions followed by recruitment of co-regulators and cofactors are the major determinants of enhancer activity and gene expression [18]. Therefore, another layer of regulation is required by controlling the accessibility of their cis-regulatory element present in the promoter-proximal or far distal enhancer regions of the target gene. The target genes that cDC1 cells express are proinflammatory cytokines (e.g. *Il12b, Il6, Il1b*, *Tnf)*, anti-inflammatory cytokines (e.g. *Il10, Socs3*) and antiviral genes such as Type I (*Ifna, Ifnb*), Type II (IFNγ) interferons, *Cxcl10, Il15,* and several interferon stimulated genes (ISGs) [19–22]. To trigger TLR-specific immune response or to maintain balance between immune response generated through multiple TLR activation, how chromatin accessibility is tightly regulated in cDC1 DCs is largely unknown [23, 24].

Co-activator or Co-repressor proteins play an important role in dynamically regulating chromatin accessibility by modifying histone proteins through either acetylases or deacetylases proteins respectively [25, 26]. For e.g., nuclear receptors co-repressors 1 (NCoR1) and silencing mediators of retinoic acid and thyroid hormone receptors (SMRT) are known to have repression activity through histone deacetylases (HDACs) proteins [27, 28]. NCoR1/SMRT were originally identified in complex of unliganded thyroid receptor and retinoic acid receptor and were thought to mediate their repression activity with only nuclear receptors but later several studies have shown the repression through other transcription factors such as BCL6, Kaiso, FOXP1 [29–32]. We have reported in our previous studies that perturbation of NCoR1 in cDC1s leads to derepression exemplified by increase in expression of proinflammatory cytokines, anti-inflammatory cytokines as well as antiviral response genes upon TLR9 activation [33, 34]. This suggests that NCoR1 mediated strong repression of genes under TLR activation is required to mount signal specific immune response. However, the epigenetic role of NCoR1-mediated change in enhancer activity in regulating immune responses generated through individual or simultaneous activation of multiple TLRs is still unknown. The NCoR1-binding sites are mostly distributed in far distal regions to the transcription start site (TSS) and were identified as repressor of PU.1 bound super enhancer in cDC1 DCs [33]. We hypothesized that NCoR1 could play an important role in regulating TLR specific enhancer activity. Genome-wide level of enhancer activity controlled by co-repressors such as NCoR1 could be measured using the H3K27ac mark that distinguishes active from inactive and poised enhancers.

To understand NCoR1-mediated regulation of enhancer activity thereby regulating TLR-specific gene expression, in this study, we investigated gene expression and enhancer activity in cDC1 under different TLR stimulation conditions. We analyzed RNA-seq and H3K27ac ChIP-seq data generated in cDC1 line (MutuDC) activated with TLR9 ligand (CpG), TLR3 ligand (pIC), and a combination of both TLR9 and TLR3 ligands (CpG + pIC). To map the signal specific active enhancers regulated through NCoR1, we also analyzed NCoR1 binding in CpG, pIC and CpG + pIC stimulation conditions using ChIP-seq data along with H3K27ac ChIP-seq and RNA-seq data in NCoR1 depleted cDC1, to understand the impact of NCoR1 on gene expression and enhancer activity after TLR activation. Based on the comprehensive analysis of the multi-omics datasets, we identified spatio-temporal activity of TLR9 and TLR3-specific enhancers showing early and late activity, respectively. Moreover, NCoR1 bound TLR9-specific enhancers showed repression exemplified by increased activity after NCoR1 knock down (KD) whereas TLR3 specifically showed decreased activity after NCoR1 KD. Also, we found that NCoR1 mediated repression of transcription could only be observed on genes upon TLR9 activation belonging to inflammatory, anti-inflammatory as well as antiviral response in cDC1. In contrast, TLR3 activation leads to decrease in antiviral gene expression. In combined TLR9 and TLR3 stimulations, cDC1 showed strong bias towards TLR9 over TLR3 both at transcription as well as enhancer activity. Furthermore, to understand the differential effect of NCoR1 in TLR3 compared to TLR9, we mapped TF ChIP-seq data based on de novo motif enrichment analysis on NCoR1 binding as well NCoR1 bound enhancers regions. We found IRF3, an important well-known SRTF under TLR3 activation, showed decreased phosphorylation as well as binding at key enhancer regions of important antiviral genes. Furthermore, the decrease in transcription of antiviral genes in pIC activated NCoR1 KD cells translated to an expected increase in Sendai Virus infection (SeV) load and decreased cytotoxic potential of CD8^+^ T-cells. Overall, our study showed that the role of NCoR1 as corepressor is biased or skewed towards TLR9 as compared to TLR3.

## Methods

### Dendritic cell culture and stimulation

The cDC1 line (MutuDC1940) was procured from Prof. Hans Acha Orbea’s lab. The group has also shown through extensive studies that the cell line resembles immature splenic murine CD8α^+^ DCs [35]. We cultured and maintained the cells in a humidified incubator at 37 degree celsius with 5% CO_2_. The MutuDC cell line carries an e-GFP reporter present at CD11c promoter.

### Generation of stable KD CD8α + line

We generated NCoR1 knock down (KD) cells using Sigma mission shRNA against NCoR1 and an Empty (Control) shRNA to generate a matched control as described [33]. We used a lentivirus mediated approach with plasmids having a pLKO.1 backbone.

Control and NCoR1 KD cells were stimulated with IFNγ, CpG-B, poly(I:C) (pIC) and combined CpG + pIC/CpG + pIC + IFNγ for 2 h or 6 h (*n* = 2). RNA-seq was performed in unstimulated, 6 h CpG, 2 h and 6 h pIC, and 6 h CpG + pIC + IFNγ stimulated condition, whereas H3K27ac ChIP-seq data were generated in unstimulated, CpG, pIC and CpG + pIC activation at 2 h and 6 h**.** The NCoR1 ChIP experiment was performed in MutuDC1940 cells in 6 h CpG, pIC and CpG + pIC + IFNγ stimulated condition.

### RNA-seq library preparation and sequencing

For RNA-seq library preparation, RNA was isolated using NEB polyA mRNA isolation kit and libraries were prepared using NEB mRNA library preparation kit. Concentrations of each sample were measured using Qubit 2.0 (Invitrogen). RNA-seq libraries were sequenced by Genotypic technology, Bangalore, India on Illumina NextSeq-500 instrument.

### RT-qPCR

For RT-qPCR, 8 × 10^5^ control cells cDC1s were stimulated with IFNγ, CpG, pIC and CpG + pIC + IFNγ for 6 h. For studies on IFNγ effect we seeded 8 × 10^5^ control cells and NCoR1 KD cDC1 and stimulated with CpG + pIC and CpG + pIC + IFNγ for 6 h. The RNA were isolated using the NucleoSpin RNA Plus miniprep kit (Machery Nagel). Total RNA isolation was carried out according to the manufacturer's protocol. RNA concentration was quantified using nanodrop spectrophotometer (Thermo). This was followed by taking 500 ng–1 μg of total RNA for cDNA preparation using high-capacity cDNA Reverse Transcriptase kit (Applied Biosystems). Quantitative PCR was performed using SYBR Green master mix (Applied Biosystems) and PCR amplification was monitored in real-time using QuantStudio-6 instrument. Primer sequence used for *Il10, Il12b, Il6, Ifnb1* has been provided in the study published previously [33]. Forward and reverse primer sequence used for *Ifit3* was 5′-CTGAAGGGGAGCGATTGATT-3′; 5′-AACGGCACATGACCAAAGAGTAGA-3′ and for *Cxcl10* is 5′-AGTGCTGCCGTCATTTTCTG-3′; 5′-ATTCTCACTGGCCCGTCAT-3′.

### Flow cytometry (FACS)

Flow cytometry analysis was carried out using a well-established intracellular (IC) staining protocol. 8 × 10^5^ cells were seeded for IC staining. Cells were either left unstimulated or were stimulated with IFNγ, CpG, pIC, CpG + pIC, and CpG + pIC + IFNγ for 6 h. Brefeldin A was added 2 h post stimulation. For staining, the cells were dissociated and washed with FACS buffer (3% FBS in 1X PBS). The cells were first fixed with 2% paraformaldehyde for 20 min followed by permeabilization using 1 × permeabilization buffer (eBiosciences). The fixed and permeabilized cells were then resuspended in IC staining buffer (FACS buffer: 1 × permeabilization buffer:: 1: 1) and stained with fluorochrome conjugated antibodies for the cytokines of interest. For optimal staining, the cells were incubated for 30–45 min in dark. After incubation, the cells were washed twice with FACS buffer and then acquired for differential expression analysis using LSRII Fortessa flow cytometer (BD Biosciences). The acquired data were analyzed using FlowJo X software (Treestar).

### NCoR1 chromatin immunoprecipitation (ChIP) and sequencing

NCoR1 ChIP assays in pIC and CpG + pIC + IFNγ stimulation conditions were performed similarly as described in our previous study [33]. For ChIP-seq library preparation, 30 μl ChIP-DNA was processed for library preparation using NEB ChIP-seq library preparation kit (Illumina). After library preparation and quality check, the libraries were sequenced by Genotypic technology, Bangalore, India on Illumina NextSeq-500 instrument.

### H3K27ac ChIP and sequencing

40 × 10^6^ Control and NCoR1 KD cells were seeded in 15 cm^2^ plates and prepared for ChIP before and after 2 h, 6 h CpG or pIC or CpG + pIC stimulation. The cells were cross-linked using 1% formaldehyde (Sigma) for 10 min at room temperature followed by quenching the reaction using 2.5 M glycine (Sigma) for 10 min. The ChIP experiments were performed as per the Mayer’s Lab Protocol. The cells were lysed in the FARHAM lysis buffer and centrifuged at 2000 rpm at 4 °C for 8 min. The chromatin was fragmented using a Bioruptor (Diagenode) sonicator for 30 min using high amplitude and 30 s ON & 30 s OFF cycles to obtain 200–500 bp size fragments. The concentration of the chromatin was estimated using a NanoDrop (Thermo) and the chromatin was diluted with a RIPA buffer prepared without protease inhibitor to make 125 μg/ml of chromatin for each IP. 30ul of Dyna Magnetic beads (Anti-rabbit) were taken and added to 1 ml tube for each IP. 3ul of rabbit monoclonal anti-H3K27ac antibody (Abcam, cat no: ab-177178), were added and incubated at 4 °C overnight on a rocker shaker. Next day, the beads were washed six to seven times with LiCl buffer (1% NP-40, 100 mM Tris HCl (pH 7.5), 500 mM LiCl, 1% Sodium Deoxycholate) followed by two washes with TE buffer (10 nM Tris HCl (pH 7.5), 0.1 mM EDTA (pH 8)). Samples tubes were pulse spinned and remaining buffers were discarded. After removing the wash buffer completely, protein-bound chromatin complexes were eluted from beads for 30 min using 200ul of elution buffers. The eluted chromatin was reverse-crosslinked by overnight incubation on the shaker using 8ul of 5 M NaCl. Next day, DNA was purified from the reverse cross-linked chromatin by proteinase-K and RNase digestion followed by purification using PCR purification kit (Qiagen). H3K27ac ChIP sample library preparation was performed using an NEB ChIP library preparation Kit and sequenced using Illumina NextSeq-550.

### RNA-seq data analysis

Raw RNA-seq fastq files were processed for quality control check using FASTQC and aligned to the mouse genome (mm10 RefSeq) using tophat2 to maintain the uniformity of analysis as unstimulated and CpG stimulated samples in control and NCoR1 KD were aligned using tophat2 in previous study. We then extracted raw counts from the respective sample using featureCounts tool (v1.6.2) [36–38]. Raw counts were then analyzed for differential gene expression analysis using DESeq2 (*v* = 1.24) [39]. Differentially expressed genes were then filtered based on log2 fold change >  = 1 and adjusted *p* value < 0.05. Normalized count and variance stabilized transformed value were used for downstream analysis. CpG specific, pIC specific and common CpG-pIC genes were identified using comparison of control CpG and pIC samples. To identify synergy/antagonist genes among CpG/pIC specific or common genes in combined CpG + pIC + IFNγ stimulation, ratio of normalized count in CpG + pIC + IFNγ and sum of individual CpG and pIC response were calculated. Genes having ratios greater than 1.2 and less than 0.5 were defined as synergy genes and antagonistic genes, respectively.

### NCoR1 ChIP-seq data analysis

Raw reads of ChIP-seq samples were processed for quality control analysis and aligned to the mouse reference genome (mm10) using bowtie2 (2.3.4.2) (with default parameter). Uniquely aligned reads were extracted (MAPQ > 10) using SAMtools [40, 41]. Peak calling were performed using findPeaks program of HOMER using style as factor and p value cut-off of 0.0001 [42]. To visualize ChIP-seq data in IGV, BigWig files were generated using the makeUCSCfile program of HOMER. Peaks were filtered against ENCODE mm10 blacklisted regions [43]. Merged peak files from all the conditions were generated using bedops (-m option). Differential NCoR1 binding sites in CpG, pIC and CpG + pIC + IFNγ were identified using the getDifferentialPeaks program of HOMER with fold change enrichment cut-off of 2 [42]. Based on fold change of enrichment, peaks were categorized into four clusters. Cluster I (twofold increase in NCoR1 enrichment in CpG, pIC and CpG + pIC + IFNγ stimulation compared to Unstimulated), Cluster II (twofold increase in NCoR1 enrichment in CpG + pIC + IFNγ stimulation compared to pIC and CpG), Cluster III (twofold increase in NCoR1 enrichment in pIC stimulation compared to CpG and CpG + pIC + IFNγ), Cluster IV (No significant change in NCoR1 enrichment across the stimulation condition), Cluster V (twofold decrease in NCoR1 enrichment after CpG, pIC and CpG + pIC + IFNγ activation). To annotate the peaks to genes, we used ChIPSeeker [44]. Peaks were annotated to its nearest genes using threshold of ± 1 kb distance from TSS. The transcript database used for the annotation is mm10 UCSC annotation (TxDb.Mmusculus.UCSC.mm10.knownGene).

### Pathway and gene set enrichment analysis

Enriched pathway terms for the gene sets from different analyses were identified using clusterProfiler R package against Reactome gene sets downloaded from MSigDB database [45]. Adjusted *p* value < 0.05 were used to filter out significantly enriched pathway terms.

### Association of DEGs with NCoR1 and H3K27ac bound targets.

Association between different gene lists were performed using GeneOverlap R package and Heatmap of log odds ratio with *p* value were plotted using complexHeatmap [46, 47].

### H3K27ac ChIP-seq data analysis

RAW single end reads were processed for quality check using the FASTQC tool and aligned to the mouse reference genome (mm10) using bowtie2 (2.3.4.2) [36, 40]. Duplicate reads were filtered using Picard MarkDuplicates (2.18.11-SNAPSHOT) and further reads were also filtered based on MAPQ cut-off < 10 [48]. MACS2 narrow peak calling program were used to call the peak in each sample against Input ChIP as background [49]. Peak summits called by macs2 in each sample were extended to ± 1 kb and overlapping peaks were merged. Consensus peak sets for H3K27ac ChIP were generated after merging 1 kb extended peak sets from each condition using bedops. To further filter down the peaks, we performed differential acetylation analysis using getDifferentialPeaks and filtered only the regions that are having twofold increase or decrease in acetylation activity after CpG, pIC and CpG + pIC stimulation. Next to perform comparison of differentially enriched H3K27ac-enriched regions across different condition we extracted raw counts using featureCounts function from Rsubread R package (1.34.7) and performed differential analysis using DESeq2(1.24.0) Genomic regions were filtered based on variance stabilized value (vst) using cut-off value of 100 (sum of vst value across all the conditions). Total differentially acetylated regions were then used to carry out Loglikehood ratio tests (lrt) in DESeq2 to get condition-specific acetylated regions. Genomics regions from clusters were merged based on condition-specific enrichment and defined into four clusters as CpG specific, pIC specific, common CpG-pIC and enhancer having decreased activity after stimulation.

### Super enhancer analysis

Super enhancer analyses were performed on macs2 called H3K27ac peaks using ROSE [50, 51]. Peaks were stitched based on the default 12 kb distance between the two peaks without exclusion of TSS. SE regions were annotated to the nearest gene using mm10 UCSC annotation from bioconductor and ChIPseeker R package [51, 44].

### Overlap of NCoR1 and H3K27ac genomics regions

Differential NCoR1-binding clusters were overlapped with differential H3K27ac-binding sites and significance of overlap were calculated using OLOGRAM (v1.2.1) [52].

### Transcription factors Motif enrichment analysis

Transcription factor motif enrichment analysis on NCoR1/H3K27ac bound genomic regions was performed using findMotifs.pl/findMotifsGenome.pl program of HOMER. Default motif lengths of 8, 10, and 12 were selected for enrichment and vertebrate options were used as known motif sets.

### Weighted gene co-expression network analysis (WGCNA)

Gene co-expression analysis of a total differentially expressed genes across comparison of samples from multiple stimulation in control and NCoR1 KD conditions were performed using WGCNA [53]. According to the method described in the WGCNA tutorial, soft power threshold was calculated using total sample and Topological overlap map (TOM) was generated. Hierarchical clustering of genes were performed based on dissimilarity of TOM and the dendrogram was cut using following parameters (minModuleSize = 30, ds = 2, cutHeight = 0.98, dthresh = 0.15) to generate co-expression modules. Pathway enrichment analyses were carried out for each module using the Reactome database from MSigDB. We identified green and darkred two important modules enriched for immune response related pathways. Out of total known TFs and coregulators (*n* = 1787) in mouse 131 were found to be significantly associated with green, darkred and salmon modules. Gene–gene interaction networks were extracted for these modules. The TFs and co-regulators were ranked in each stimulation condition based on significance of association of identified target from gene–gene interaction network and the target differentially expressed in each condition. Furthermore, known protein–protein interactions of identified gene–gene co-expression networks were validated using StringDB in Cytoscape (V.3.7.1) [54, 55].

### ChIP-seq analysis of publicly available datasets

SRA files of transcription factor PU.1, JunB, cRel, IRF3 ChIP-seq data at 0 h, 90 min CpG and pIC performed in the MutuDC1940 (GSE106730) and PU.1, IRF1, IRF4, RelA, RelB, Rel, JunB, Stat1 and Stat3 ChIP-seq data at 0 h and LPS stimulation performed in bone marrow derived dendritic cells (GSE36104) were downloaded from NCBI Gene Expression Omnibus. Raw fastq files were extracted using the fastq-dump program of SRA Toolkit (2.9.2) [56]. Reads were aligned to mouse reference genome mm10 using bowtie2 (2.3.4.2) and reads having mapping quality (MAPQ) < 10 were filtered out to carry out downstream analysis. Peak calling for mutuDC cell line ChIP data was performed using findPeak function of HOMER. Peaks were filtered against ENCODE mm10 blacklisted regions [43]. Genomic regions for each TF data identified in mutuDC were overlapped with NCoR1 genomic regions overlapping with H3K27ac as well as associated with CpG/pIC or common CpG-pIC-specific genes using bedtools. The bedGraph file for each ChIP-seq data was generated using the makeUCSCfile program of HOMER. TFs/H3K27ac Enrichment heatmap ± 2 kb to NCoR1 peak center were generated using deepTools2 (3.5.1) [57].

### Western blots

Empty and NCoR1 KD DC line were plated at 2x10^6^ in each well of 6 well plate and treated with poly I:C at 5ug/ml (invivogen TLRL-pic-5) and CpG ODN at 1ug/ml (invivogen 1826) for 2 and 6 h separately, followed by lysis in RIPA buffer (0.5 M EDTA, 1 M Tris–Cl pH7.5, 1 M NaCl, 200 mM PMSF, 10% NP-40, 10% SDS, 5% sodium deoxycholate, 1 M sodium orthovanadate and 1X Roche protease inhibitor). Cells were sonicated in Bioruptor (Diagenode) with setting of high amplitude and 30 s ON and 30 s OFF for ten cycles. After complete lysis, samples were processed for protein quantification by BCA protein assay kit (Bio-Rad). We loaded the samples at 50–80 ug concentration and SDS-PAGE was performed, either in 10% gel for IRF3 or 15% gel for ISG-15, at 80–100 V. Furthermore, we transferred the gel onto a nitrocellulose membrane and probed with phospho-IRF3 (cst 29047S) or ISG-15 (sc166755) or tubulin (cst 2146S). Once p-IRF3 was developed we stripped the blot and reprobed the same blot with total-IRF3 (cst 4302S). Finally, once again the blot was stripped and probed with loading control β-tubulin. We developed the blot on BioRad Chemidoc. Densitometric analysis was performed using ImageJ software.

### IRF3 ChIP and qPCR

ChIP for IRF3 was performed according to a well-established method used by Raghav and Deplanke’s lab [32]. For performing ChIP assays, we seeded 40x10^6^ cells in 150 mm × 25 mm petridishes. The cells were either left unstimulated or stimulated with polyIC at 5 μg/ml (invivogen TLRL-pic-5) for 2 h. Cells were then crosslinked with 1% formaldehyde (Sigma 252,549) at room temperature for 10 min and then the reaction was quenched using 2.5 M glycine (Sigma 50,046) for 5 min at room temperature. The petridishes were then placed on ice and cells were scraped using 1X PBS and collected in falcon tubes. The tubes were centrifuged and pellets were washed twice with chilled 1X PBS. Finally, the pellets were stored in -80 degrees for future use.

On the day of performing the ChIP experiment, pellets were taken out and thawed on ice. The cell pellet was then subjected to lysis using Nuclear extraction buffer (Hepes–KOH pH7.5, NaCl, EDTA pH 8.0, glycerol, NP-40, triton-X supplemented with protease and phosphatase inhibitors) for 10 min at 4 degree with constant mild shaking. The cells were then centrifuged at 2500 rpm for 5 min and pellets collected. Next, the isolated nuclei were subjected to a protein extraction buffer (NaCl, EDTA, Tris–Cl pH 8.0, supplemented with protease and phosphatase inhibitors) for 10 min at room temperature with constant mild shaking. The tubes were centrifuged and pellets collected. Finally the nuclei were then subjected to chromatin extraction buffer (EDTA, Tris–HCl pH 8.0, triton-X supplemented with protease and phosphatase inhibitors) and incubated for 10 min on ice. The extract was then sonicated using bioruptor (diagenode) with the following settings: 30 s on, 30 s off, 35–40 cycles. Once the desired fragment size was obtained (200–400 bp) we quantified the chromatin and 150ug chromatin was used per ChIP. The chromatin was resuspended in ChIP dilution buffer (EDTA, TriS–HCl pH 8.0, triton-X, NaCl supplemented with protease and phosphatase inhibitors). 1% input was kept separately in this step.

BSA blocked recA-sepharose beads (invitrogen 101,142) was used for pull down. The pre-blocked sepharose beads were used 80 ul/IP and incubated with chromatin for 2 h at 4 degree with rotation for any non-specific chromatin removal. The beads were then centrifuged and the unbound supernatant was then incubated with 5 ul of total-IRF3 (cst 4302 s) and mAb IgG rabbit (cst 3900 s) overnight. Next day, the chromatin bound antibody complex was incubated with BSA pre-blocked sepharose beads for pulling down the bound complex for 2 h. After 2 h incubation, the tubes were centrifuged and supernatant discarded. The pellet was then washed with the following buffers for twice each: low salt buffer (Tris–HCl pH 8.0, NaCl, EDTA, SDS, triton-X), high salt buffer (Tris–HCl, NaCl, EDTA, SDS, triton-X), lithium chloride wash buffer (Tris–HCl pH 8.0, LiCl, EDTA, NP-40, sodium deoxycholate), and TE wash buffer (Tris–HCl and EDTA). Finally the chromatin was eluted in an elution buffer (sodium bicarbonate, SDS) and eluted from beads by constant shaking at room temperature for 15 min. The tubes were then centrifuged and eluted supernatant was collected. The supernatant was reverse crosslinked using NaCl overnight with constant shaking at 65 degrees. Next day, the reverse crosslinked chromatin was subjected to proteinase-K and RNase treatment and finally PCR purified using Qiagen PCR purification kit (Qiagen 28,006).

For experimental validation of IRF ChIP, ChIP-qPCR was performed at 0 h and 2 h pIC activated control and NCoR1 KD DCs. Enrichment of these factors at randomly selected ChIP-seq positive genomic regions/genes was calculated in comparison to negative control genomic regions. Three independent ChIP experiments were performed for IRF3 ChIP-qPCR. Fold enrichment at positive genomic regions was calculated relative to negative control regions. The ChIP primers used are listed in the table below. The *p* value for enrichment significance was calculated using two-tailed paired Student’s *t* test and error bars depicted SEM in the fold change error in enrichments observed in different biological replicates.

Table showing list of sequence of primer used for IRF3 qRT-PCR

**Table:** 

Target	Forward	Reverse
Negative Control	AGTGGTCAGTGCCAAGTTCA	CACCCCAAGGCTACAGTCAT
Ifnb1	GCTACCTGCAAGATGAGGCA	GAGGCAGAAAGGACCATCCC
Isg15	GTGAAGAGGCGGAGTTTCCA	GAGCCAGTCCCTTTCCTTCC
Cxcl10	CCCTGAGTCCTGATTGGCTG	AATGCCCTCGGTTTACAGGG
Il15	AAGGCACAAGGAGCGAATCA	GTTAGCTGGGGTTGGGACTC

### IFNβ ELISA

ELISA was performed to estimate the IFNβ levels secreted in the cell culture supernatants according to the manufacturer’s protocol (ab25263). Briefly, supernatants were collected from control and NCoR1 depleted cDC1 after 2 and 6 h of pIC stimulation and stored at − 80 °C in small aliquots until analysis. The supernatants were diluted to 1:4 using sample diluent and then used for the assay. All standards and samples were assayed in duplicates.

### Sendai virus (SeV) infection in DCs

Control and NCoR1 KD CD8α ^+^ DCs were seeded in a flat bottom 96-well plate at a density of 4 × 10^4^ cells/well. Cells were left overnight for acclimatization and proper adherence. Next day, cells were stimulated with TLR3 specific synthetic ligand polyI:C (pIC) at 5 μg/ml for 6 h at different dilutions. After pre-incubation, SeV-tomato red infection was carried out for an additional 16 h. Percent SeV infection and MFI was represented in the flow cytometer at 594 nm emission wavelength.

### Co-culture of DCs with CD8 + T-cells for assessing T-cell proliferation and cytotoxicity

DC-T-cell co-culture experiments were performed according to well established protocol [58–60]. Naïve CD8 ^+^ T-cells were purified from the spleen of TCR-transgenic OT-I mice using CD8 ^+^ T-cell isolation kit. NCoR1 KD and control cDC1 were seeded at a density of 10,000 cells/well in round bottom 96 well plates followed by pulsing with OVA peptide (257–264) /OT-I at 5 nM concentration overnight. Further, DCs were stimulated with 5 μg/ml of pIC for 6 h at different dilution (1:1, 1:10 and 1:100). After 6 h, media were aspirated and fresh media containing purified OT-I T-cells were added at the density of 100,000 cells/well. Then, T-cell proliferation and cytotoxicity of T-cells were analyzed by FACS. Proliferation was measured using an amine-based dye (eFluor 670). The rate of T-cell proliferation was inversely proportional to the median fluorescence intensity (MFI) measured in FACS after 72 h of co-culture. For cytotoxic T-cell differentiation profiling after 96 h, the co-cultured T-cells were re-stimulated with PMA (10 ng/mL), ionomycin (500 ng/mL) and Brefeldin-A (10 μg/mL) for 5 h. Fluorochrome conjugated antibodies specific to cytotoxic T-cell (Perforin [eBioscience:12–9392-80], IFN-γ [eBioscience:25–7311-41], Granzyme-B [Biolegend:515405]) were checked in CD3^+^ CD8^+^ CD44^+^ [eBioscience:48–0441-82] effector T-cells using respective fluorescence minus one (FMO) controls.

## Results

### RNA-seq of TLR9, TLR3 and combined TLR9 + TLR3 activation reveals immune response signatures genes in cDC1

**Fig. 1:**
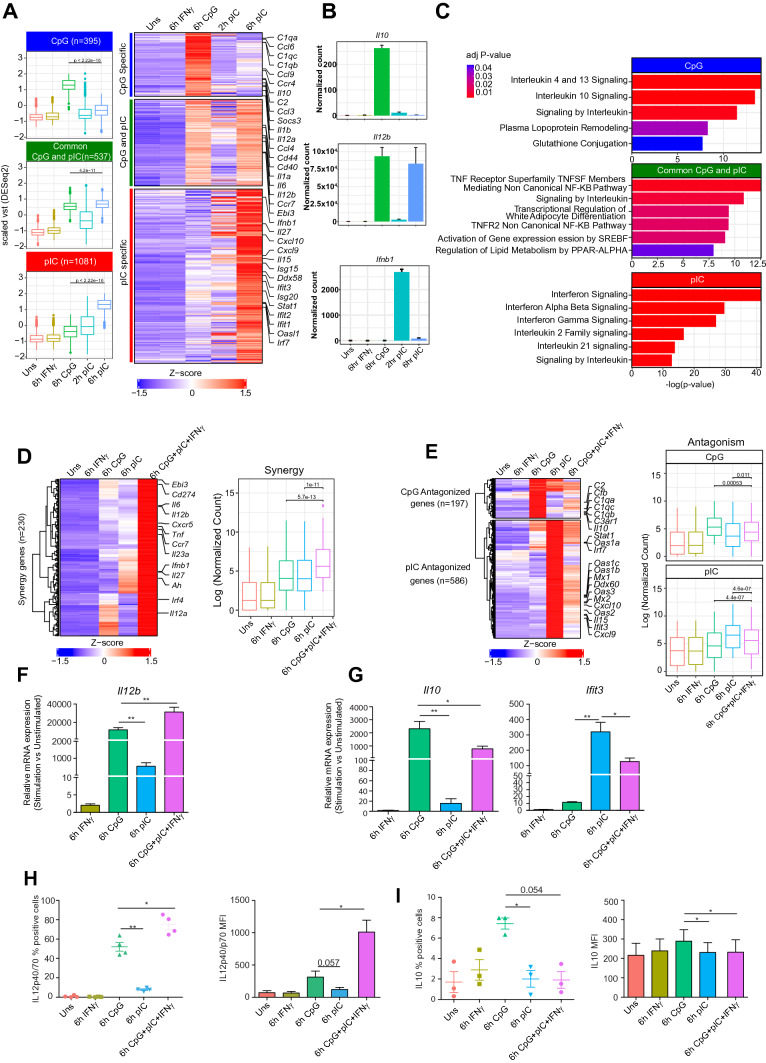
Gene expression analysis of unstimulated and 6 h (CpG, pIC and CpG + pIC) stimulated DCs using RNA-seq. **A **Boxplot (1st panel) and Heatmap (2nd panel) showing scaled variance stabilized transformed (vst) values from DESeq2 of CpG, pIC specific and common CpG-pIC genes identified from gene expression analysis. Important DC response genes specific to CpG and pIC are marked on the right side of the heatmap. Gene expression values have been scaled with mean and standard deviation across the condition. **B **Bar plot showing DESeq2 normalized count of an exemplary CpG, pIC specific and common CpG-pIC gene. **C **Plot showing significantly top enriched Reactome term from MSigDb database for CpG, pIC specific and common CpG-pIC gene sets in cDC1. **D** Heatmap (1st panel) representing the expression pattern of the genes depicting synergy in transcript expression upon combined stimulation with CpG + pIC. Boxplot (2nd panel) showing distribution of the log normalized count values from DESeq2. **E **Heatmap ( 1st panel) representing the expression pattern of the genes showing antagonistic effects upon combined CpG + pIC stimulation of cDC1. Boxplot (2nd panel) showing distribution of the normalized vst values from DESeq2. **F** RT-qPCR showing relative mRNA expression of *Il12b* before and after TLR/IFNγ stimulation in control cDC1 (*n* = 6). **G** RT-qPCR showing relative mRNA expression of *Il10* and *Ifit3* before and after TLR/IFNγ stimulation in control cDC1 (*n* = 6). **H** Scatter dot plot and bar plot representing percent positive cells and MFI respectively for IL-12p40/p70 (*n* = 4). **I** Scatter dot plot and bar plot representing percent positive cells and MFI respectively for IL-10 (*n* = 3). The Wilcoxon test was used to calculate the significance of the difference between the mean for Fig. 1A, D (2nd panel) and 1F (2nd panel). Two tailed paired student’s t-test was used to calculate p-value (*p ≤ 0.05, **p ≤ 0.01 and ***p ≤ 0.001). Data shown in Fig. 1F–I is combined from 3 or more than 3 independent experiments

It has been well characterized by us and others that TLR9 and TLR3 are majorly expressed in murine cDC1, where TLR3 ligation by poly I:C (pIC) results in an antiviral response and TLR9 ligation by CpG results into strong inflammatory response [33, 61]. To profile the global gene expression changes and identify the signature genes specific to TLR9 and TLR3, we analyzed global transcriptome changes in mouse cDC1 (CD8α^+^ MutuDC) line treated with CpG-B (TLR9) and pIC (TLR3) ligand for 6 h. As there are reports that antiviral responses in dendritic cells are early responses mediated by major type-I interferon gene *Ifnb1,* we also analyzed transcriptome at 2 h in pIC activation [61]. We also used individual IFNγ stimulation as host response factor and combined CpG + pIC + IFNγ (TLR9 + TLR3 + IFNGR) stimulation to understand synergistic and antagonistic activity in cDC1. Principal component analysis (PCA) separated unstimulated and TLR activated cDC1s, however, IFNγ stimulated sample clustered with unstimulated (Supplementary Fig. 1A). Next, we performed differential gene expression analysis to identify the effect of IFNγ, CpG, and pIC. As reported in other studies, IFNγ alone does not have an impact on gene expression and only slightly increases Il12b expression [62]. We also observed that only 7 genes are differentially regulated in IFNγ stimulation in cDC1 including *Il12b* which showed only a slight increase compared to unstimulated (Supplementary Fig. 1B, C). Next, out of a total 4829 differentially expressed genes (DEGs) (log2 fold change >  = 1 or <  = -1 and adjusted p value < 0.05) upon 6 h CpG or pIC activation, we identified 395 genes expressed specifically in CpG condition, 1081 in pIC and 537 commonly expressed in both CpG and pIC stimulation condition (Fig. 1A, Table S1). CpG stimulation led to expression of inflammatory cytokines such as *Il1b, Il12a* and also a few anti-inflammatory genes such as *Il10* and *Socs3.* Major pro-inflammatory genes such as *Il12b, Il6* are expressed in both CpG and pIC activation at 6 h whereas antiviral responses such as *Ifnb1* and *Cxcl10* showed expression at the early 2 h time point upon pIC activation (Fig. 1A, B)*.* The pathway enrichment analysis of these corresponding gene-sets against reactome database showed significant enrichment of inflammatory and anti-inflammatory pathways (IL4 and IL13 signaling, IL10 signaling) for CpG-specific genes, NFkB signaling for common genes and Interferon signaling for pIC-specific genes (Fig. 1C). Several studies involving co-infection of DCs with both bacteria (e.g. *Mycobacterium tuberculosis*) and viruses (e.g. HIV) have shown to decrease antigen presentation through MHC-II or decrease in co-stimulatory molecules expression [63, 64]. Moreover, simultaneous activation of multiple TLRs has also been shown to have synergistic and antagonistic effects on the gene expression [39–69]. To understand the synergy and antagonistic activity in cDC1, first, we checked the effect of all three stimuli (CpG + pIC + IFNγ) with combined TLR activation (CpG + pIC) on several TLR response genes. We observed no significant difference on major TLR response genes between CpG + pIC + IFNγ vs CpG + pIC activation (Supplementary Fig. 1D). Next we identified synergistic and antagonistic effects of TLR9 and TLR3 activation on gene expression from RNA-seq data in combined CpG + pIC + IFNγ stimulated DCs. We defined synergy genes as the ones that depicted at least 1.2-fold transcript expression in combined stimulation as compared to the sum of expression in individual CpG and pIC activation conditions (see “Methods” for details). We found 230 genes showing synergy including CpG-specific genes (e.g., *Il12a, Irf4*), pIC specific genes (e.g. *Ifnb1, Il27*) and CpG-pIC common genes (e.g. *Il12b, Il6*) (Fig. 1D). Similarly, we define the antagonist effect, if the ratio of gene expression in combined stimulation and sum of individual (CpG/pIC) activation is less than 0.5 (see “Methods” for details). We found a large number of pIC specific genes (e.g. *Cxcl10, Il15, Irf7, Ifit3, Mx1, Mx2*) (n = 586) and few CpG specific genes (e.g. *Il10, C1qa, C1qb, C1qc, C3ar1*) (*n* = 197) showing antagonistic regulation upon combined CpG + pIC + IFNγ stimulation (Fig. 1E, Supplementary Fig. 1E, Table S1). To check the synergy and antagonist behavior in combined stimulation, we validated the expression at transcript and protein expression using RT-qPCR and flow cytometry method respectively. RT-qPCR analysis showed synergy in *Il12b* and antagonism in *Il10* and *Ifit3* expression at transcription level (Fig. 1F, G). Further flow cytometry analysis also confirmed synergy and antagonistic activity in IL12b and IL10 expression at protein levels, respectively (Fig. 1H, I). However, no significant difference could be observed between CpG + pIC and CpG + pIC + IFNγ activated control cDC1 at both mRNA and protein (Supplementary Fig. 1F, H). Corroborating with previous studies on synergistic or antagonistic activity of two different TLRs, cDC1s show synergistic and antagonistic activity on gene expression upon combined TLR9 and TLR3 stimulation. TLR9 and TLR3 ligation resulting in synergistic expression of immune response genes as well as suppression of a large number of effector ISGs and anti-inflammatory cytokine (*Il10*) in combined stimulation condition suggests an interesting role of TLR9 or TLR3-specific enhancer activity that in turn is regulated through co-repressor proteins such as NCoR1 in fine-tuning the underlying gene expression.

### Differential spatio-temporal regulation of enhancer activity upon TLR9 and TLR3 ligation in cDC1

**Fig. 2:**
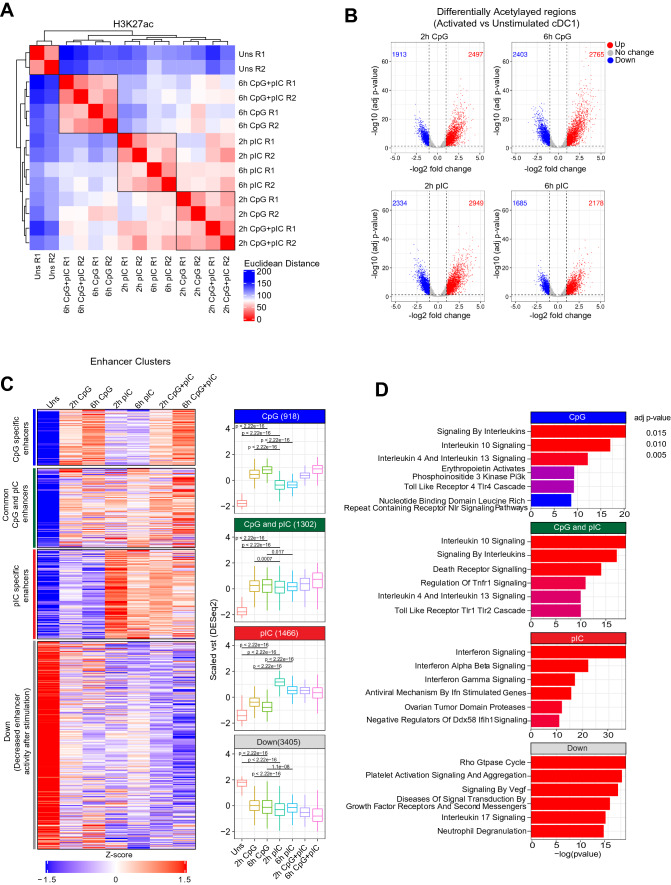
Global profiling of H3K27ac histone marks in unstimulated, 2 h and 6 h CpG, pIC and CpG + pIC stimulated cDC1 identified TLR specific active enhancers. **A** Clustering of H3K27ac marks from two independent biological replicates of unstimulated, 2 h, and 6 h CpG, pIC and combined CpG + pIC stimulated cDC1 based on euclidean distance. **B** Volcano plot showing differential H3K27ac genomic regions in cDC1 cells after 2 h, and 6 h CpG, and pIC activation as compared to unstimulated condition. **C** Heatmap (1st panel) representing CpG, pIC and combined CpG + pIC stimulation specific enhancers. Variance stabilized transformed (vst) values were scaled using mean and standard deviation across the samples. Boxplot (2nd panel) representing distribution of scaled vst of H3K27ac enrichment in different conditions. Wilcoxon tests were carried out to compare the difference in mean and to calculate the p value significance between the conditions. **D** Barplot depicting the enriched reactome pathway from MSigDB database enriched in the activation specific enhancer clusters showing stimulation dependent enhancer activity

Enhancer activity plays a significant role in determining cell type or condition specific gene expression and always highly correlates with gene expression [70, 71]. Posttranslational modifications at 27th lysine residue of H3 protein are known to identify active enhancers either at promoter-proximal or far distal regulatory regions [72, 73]. To profile the TLR9, TLR3 and combined TLR9 + TLR3 stimulation-specific enhancers controlling the downstream target genes and their dynamic temporal activity, we performed H3K27ac ChIP-seq in the cDC1 in unstimulated, 2 h and 6 h stimulated (pIC, CpG and CpG + pIC) conditions. We identified a total of 40–48 K ChIP-seq peaks depicting H3K27ac marked genomic regions across different conditions in comparison to Input. Based on cut-off on variance stabilized transformed (vst) value and differential enrichment of ChIP-seq peaks calculated using HOMER in pIC, CpG and CpG + pIC stimulation conditions, we filtered out total H3K27ac peaks to 11,750 (see “Methods” for details). Hierarchical clustering analysis of the H3K27ac samples based on euclidean distance of the biological replicates in respective conditions showed that all the replicates in respective conditions clustered together (Fig. 2A). The clustering analysis also showed that CpG stimulated samples at 2 h and 6 h clustered more closely with 2 h and 6 h CpG + pIC stimulated samples, while 2 h and 6 h pIC samples clustered together suggesting combined stimulation effects are dominated by CpG challenge (Fig. 2A). To further identify the temporal enhancer activities in different conditions, we performed differential enhancer enrichment analysis on 11,750 genomic regions. We found 6 h CpG samples had relatively more number of genomic regions showing differential enrichment compared to 2 h CpG and vice-versa for pIC stimulation (Fig. 2B), whereas combined stimulation with CpG + pIC showed a comparable number enhancer regions showing increased or decreased activity compared to unstimulated (Supplementary Fig. 2A). Next, to identify the CpG and pIC-specific enhancers, we performed lrt (log likelihood ratio test) using DESeq2 to identify significant variable enhancer activity across the different stimulation conditions and time points. We identified 7091 genomic regions showing significant variability in enrichment across different conditions. Then, we grouped the H3K27ac peaks using hierarchical clustering approach based on pairwise correlation of vst value of H3K27ac enrichment obtained from DESeq2. Broadly we identified four clusters, CpG specific (*n* = 918), pIC specific (*n* = 1466), common CpG-pIC (*n* = 1302) and down cluster (cluster having decreased enhancer activity after TLR stimulation) (*n* = 3405) (Fig. 2C, Table S2). Differential H3K27ac enrichment analysis suggested an early enhancer activity on pIC-specific enhancer at 2 h in pIC that decreases further at 6 h, whereas CpG specific enhancers showed a delayed and sustained dominance on enhancer activity till 6 h (Fig. 2C). After we identified TLR-specific enhancers showing activity in time dependent manner, we annotated these genomic regions to nearest genes and performed pathway enrichment analysis. CpG, pIC and common CpG-pIC enhancer clusters were found to be enriched for immune response pathways (Fig. 2D). Overall, differential H3K27ac enrichment analysis suggests that cDC1 shows differential spatio-temporal enhancer activity upon TLR9 and TLR3 activation.

### Enhancer activity correlates strongly with signal specific immune-response gene expression upon TLR9 and TLR3 ligation

**Fig. 3:**
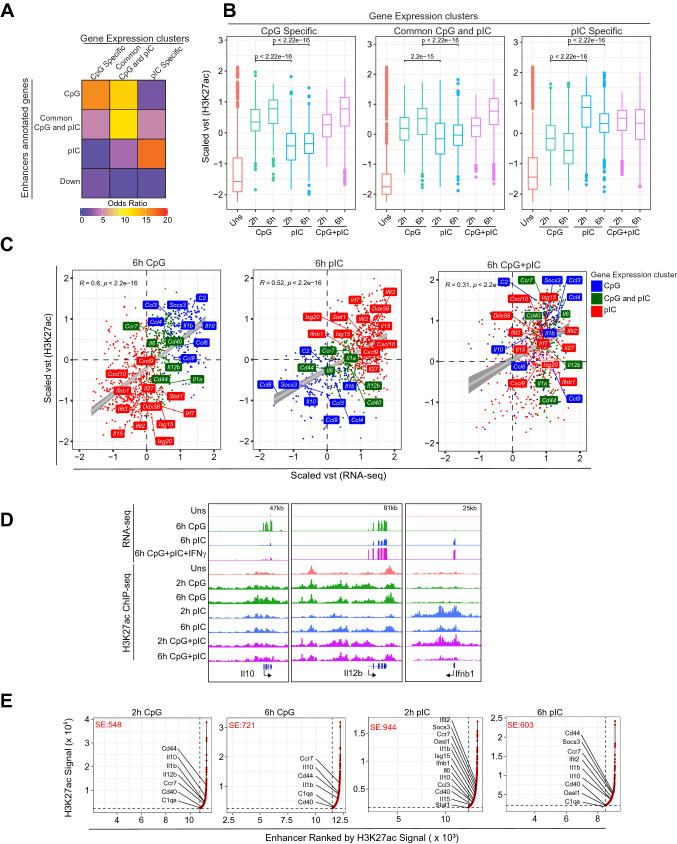
Integration of stimulation specific H3K27ac enhancers with global gene expression data in cDC1. **A** Heatmap showing odds ratio of association of CpG, pIC specific or common CpG-pIC genes with genes annotated to CpG and pIC specific enhancer. **B** Boxplot showing the scaled vst comparison of H3K27ac ChIP enrichment on CpG, pIC and common CpG-pIC specific genes identified in Fig. 1B. **C** Scatter plots depicting the correlation of gene expression and H3K27ac enhancer activity in CpG, pIC and CpG + pIC stimulated condition. The CpG, pIC specific and common CpG-pIC genes are highlighted in blue, red and green color respectively and selected representative genes from respective clusters are marked in the plots. **D** IGV browser snapshot representing RNA-seq and H3K27ac enrichment in unstimulated, CpG, pIC and combined CpG + pIC on *Il12b*, *Il6*, *Il10* and *Ifnb1* gene loci. **E** Scatter plots showing the Super Enhancers identified at 2 h and 6 h (CpG, pIC) stimulated cDC1. The CpG/pIC specific genes annotated to the Super Enhancer regions have been marked for each condition

After identifying the TLR9 and TLR3-specific enhancers, we associated the genes annotated to different enhancer clusters with gene expression clusters identified based on differential expression of 6 h RNA-seq data as CpG specific, pIC specific and common CpG-pIC response genes. We found significant association in terms of odds ratio for CpG specific (*p* = 5*e *− 60), pIC specific (*p* = 2*e *− 235) as well as common CpG-pIC response genes (*p* = 5*e *− 77) (Fig. 3A). Moreover CpG specific enhancers also showed significantly higher association with common CpG-pIC response genes (Fig. 3A, B). Furthermore, we also correlated enhancer activity and gene expression of CpG specific, pIC specific and common CpG-pIC response genes in each condition. Interestingly, we found positive correlation between gene expression and enhancer activity in each CpG, pIC and combined (CpG + pIC) stimulated condition (Fig. 3C). We coloured the dots (genes) in the scatter plot as CpG specific and pIC specific and common CpG-pIC genes and mark a few genes in each category belonging to inflammatory, anti-inflammatory and antiviral response (Fig. 3C). Inflammatory genes such as *Il12b, Il6* showed increase in enhancer activity and expression in CpG, pIC and CpG + pIC activation condition while anti-inflammatory genes such as *Il10* and *Socs3* showed an increase in only CpG and CpG + pIC activation condition (Fig. 3C, D). On the other hand, antiviral genes (*Ifnb1, Cxcl10*) and other effector ISGs (*Ifit3, Isg15*) showed increased enhancer activity and expression in pIC and CpG + pIC activation condition (Fig. 3C, D). Moreover, as we observed early enhancer activity at 2 h in pIC stimulated conditions, we further checked if pIC-specific genes follow a similar trend at expression level. Interestingly, we observed similar trends in both enhancer activity and gene expression on several pIC specific genes (Supplementary Fig. 2B). Furthermore, as we observed synergistic and antagonistic activity of CpG and pIC specific genes, we sought to look into if the enhancer activity also behaved similarly. Interestingly, we found similar synergistic and antagonistic patterns on enhancer activities in CpG + pIC activation condition (Supplementary Fig. 2C). We also identified super-enhancer (SE) regions based on H3K27ac peaks associated with CpG and pIC-specific genes using the ROSE program [50, 51]. Higher number of SE activity at 6 h CpG activation compared to 2 h and vice-versa for pIC activation further confirms late and early regulation of gene expression in CpG and pIC activation, respectively. Though 2 h and 6 h combined CpG + pIC activation showed a comparable number of SE regions, however, only 2 h showed SE activity for major TLR response genes which is reduced at the later 6 h time point for antiviral response genes such as *Ifnb1*, *Isg15, Ifit2* etc. (Fig. 3E, Supplementary Fig. 2D). The temporal change in TLR9 and TLR3 specific enhancer activity on CpG and pIC specific genes, respectively, and their associated SE regions clearly indicated the role of identified enhancers in regulating signal specific gene expression in time-dependent manner. Moreover, the synergistic and antagonistic enhancer activities substantiated the TLR9 dominance on enhancer activity in combined CpG + pIC stimulation.

### NCoR1 binding regulates TLR9 and TLR3 specific enhancer activity in cDC1

**Fig. 4:**
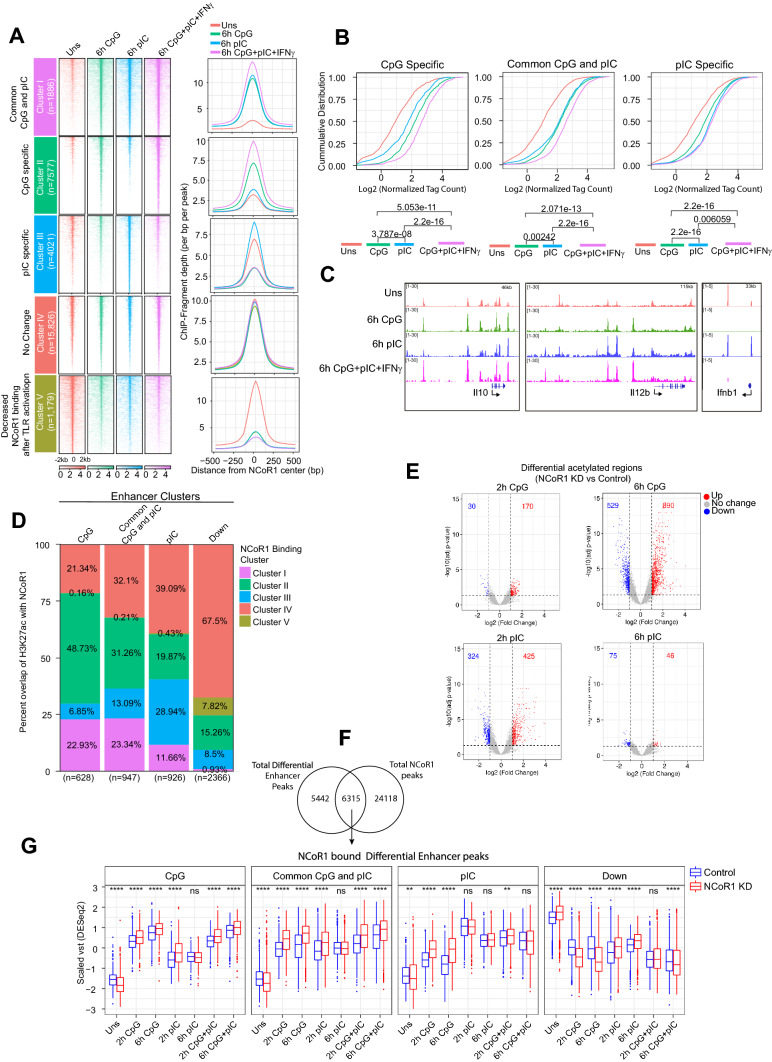
Stimulation specific NCoR1 ChIPseq binding in CpG, pIC and combined CpG + pIC specific condition and impact of NCoR1 depletion on stimulation specific H3K27ac enhancer activity. **A** NCoR1 ChIP-seq enrichment heatmap plots (1st panel) representing NCoR1 bound peaks (± 2 kb of peak maxima) in unstimulated and 6 h CpG, 6 h pIC and combined 6 h CpG + pIC stimulated conditions for each identified cluster. Normalized coverage (Reads per base per peak) density plot showing enrichment of NCoR1 for each cluster. **B** Density plot showing empirical cumulative distribution of NCoR1 binding in unstimulated, 6 h CpG, 6 h pIC and combined 6 h CpG + pIC challenged condition in cDC1. Kolmogorov–Smirnov test were performed to calculate the significance of difference in the 6 h enrichment. **C **IGV browser snapshot representing NCoR1 binding in unstimulated, CpG, pIC and combined CpG + pIC+ IFNγ on *Il10, Il12b* and *Ifnb1* gene loci. **D** Stacked bar plot showing percent overlap of H3K27ac binding sites in control cDC1 with NCoR1 binding clusters shown in Fig. 4A. Number below the bar plot represents the total number of genomic regions depicted overlap with each enhancer cluster. **E** Volcano plot showing total number of differentially acetylated genomic regions after NCoR1 depletion in 2 h, and 6 h (CpG and pIC) stimulated conditions compared to control cDC1. **F** Venn diagram showing overlap of total NCoR1 peaks and differential enhancer peaks. **G** Boxplot showing distribution of enhancer activity of NCoR1 bound differential enhancer in control and NCoR1 KD CpG, pIC and CpG + pIC activated cDC1. Wilcoxon test was performed to compare the mean between Control and NCoR1 KD (ns: *p* > 0.05, *p  ≤  0.05, **p  ≤  0.01, ***p <  = 0.001,****p  ≤  0.0001)

We recently reported that NCoR1 acts as a direct repressor of tolerogenic and antiviral immune response genes upon TLR9 ligation in cDC1 [33, 34]. TLR9 activated NCoR1 depleted cDC1 showed increased *Il10* and *Ifnb1* gene expression. Here, we further investigated the role of NCoR1 in regulating TLR9 and TLR3-specific enhancer activity and thereby underlying gene expression. First, we did comprehensive analysis and comparison of NCoR1 bindings across 6 h CpG, pIC and the combined CpG + pIC + IFNγ stimulation. We identified a slightly increased number of binding sites in pIC and CpG + pIC compared to unstimulated and CpG stimulation conditions (Supplementary Fig. 2E). Similar to unstimulated and CpG stimulation, we found more than 85% of the binding sites are distributed in the far distal regions based on distance relative to TSS in case of pIC and CpG + pIC stimulation (Supplementary Fig. 2F). To further understand stimulation-dependent NCoR1-mediated gene regulation, we compared the NCoR1 bindings in unstimulated with 6 h challenge (pIC, CpG and CpG + pIC + IFNγ) conditions to identify differential NCoR1 binding sites. Based on the fold-change of NCoR1 bindings in comparison to unstimulated cDC1, we identified five clusters of genomic regions depicting differential binding intensity of NCoR1 across the stimulation conditions. A large number of NCoR1-binding sites (Cluster IV; *n* = 15,826) were identified that did not show any significant change across the different stimulation conditions whereas few sites (Cluster V; *n* = 1179) showed decreased NCoR1 binding after TLR ligation (Fig. 4A, Table S3). Other three sets showed an activation-dependent increase in NCoR1 binding. Only a small fraction of genomic regions (Cluster I; *n* = 1886) showed an increased NCoR1 binding after CpG, pIC and CpG + pIC + IFNγ stimulation compared to unstimulated. On the other hand, 7577 genomic regions depicted in Cluster II showed an increase in NCoR1 binding upon CpG ligation, which is further increased in combined CpG + pIC + IFNγ stimulation. In pIC challenged conditions, 4021 genomic regions (Cluster III) showed significantly increased NCoR1 binding as compared to CpG and CpG + pIC + IFNγ stimulation (Fig. 4A, Table S3). As evident from the large number of NCoR1-bindings sites showing increase in binding enrichment after CpG stimulation (Cluster I and Cluster II), cDC1 appears to recruit NCoR1 on a large number of regulated genes upon TLR9 ligation compared to TLR3. Next to understand how increase in NCoR1 binding after cDC1 stimulation regulates CpG/pIC specific response genes, we associated the NCoR1-binding clusters with TLR9 and TLR3 response genes from RNA-seq data. We found a significant association of condition specific NCoR1 bindings with condition specific gene expression and are enriched for immune response pathways (Fig. 4B, C, Supplementary Fig. 2G, H). However, there are NCoR1-binding sites that do not show significant change in enrichment at the genes that are expressed in a TLR stimulation-specific manner (Supplementary Fig. 2H). We found that there are multiple regulatory regions nearest to the genes with different levels of NCoR1-binding enrichment on TLR-specific genes that could dynamically control TLR-specific enhancer activity and thereby control the gene expression in a TLR-dependent manner. NCoR1 co-repressor complex is known to control the enhancer activities, so to analyze that, first we performed correlation of stimulation specific NCoR1-binding changes with H3K27ac enhancer activity. Interestingly, we found that change in NCoR1-binding enrichment strongly correlates with change in H3K27ac intensity near ± 500 bp to NCoR1 peak center for each of the stimulation conditions (Supplementary Fig. 3A). Furthermore, we observed significant overlap of the TLR-dependent NCoR1-binding cluster showing increase in binding with the TLR dependent increased H3K27ac-binding cluster (Fig. 4D and Supplementary Fig. 3B). The association of signal specific increased enhancer activity with increased NCoR1 binding at 6 h activated cDC1 was surprising as co-repressor binding is known for deacetylase activity through HDAC3 [74]. As switch between co-repressor and co-activator binding may occur cyclically, hence clearance of NCoR1 on enhancer regions might be also a temporal event and captured through ChIP-seq of NCoR1 temporally [75]. Further to identify the impact of NCoR1 depletion on enhancer activity, we analyzed H3K27ac enhancer activities in NCoR1 KD unstimulated, CpG, pIC and CpG + pIC stimulation conditions. The increased enhancer activity after NCoR1 depletion was observed at 6 h in CpG and 2 h in pIC simulations (Fig. 4E and Supplementary Fig. 3C, D). This again strongly supports our observation of late and early induced enhancer activities in case of CpG and pIC, respectively. Next, to identify the effect of NCoR1 KD on CpG/pIC-specific enhancers, we looked into H3K27ac enrichment in control and NCoR1 KD on NCoR1 bound differential enhancers (Fig. 4F, G). Increased H3K27ac enhancer activity on NCoR1 KD CpG activated condition and pIC showed not significant, however, decrease in trend suggest NCoR1 represses enhancers activity in TLR9 but not in TLR3 activation in cDC1.

### NCoR1 KD-mediated gene regulation is biased towards TLR9 versus TLR3

**Fig. 5:**
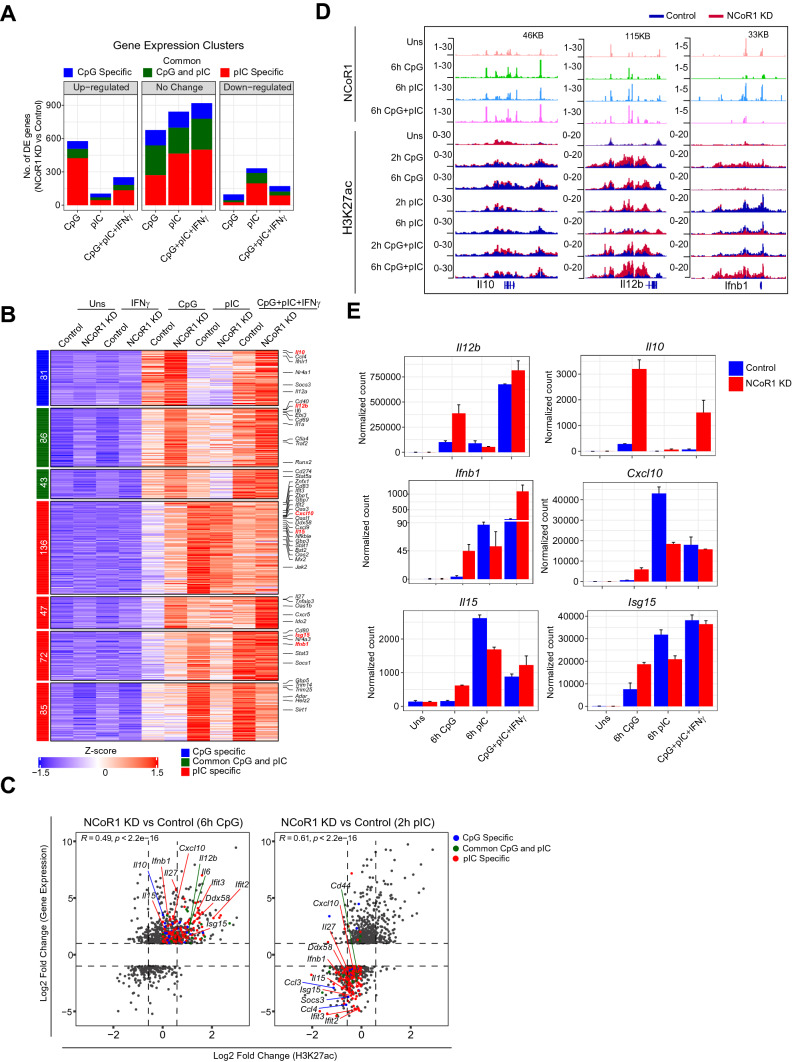
Integrative analysis of global scale gene expression, enhancer activity, and NCoR1 binding depicting NCoR1 control on signal specific cDC1 immune responses. **A **Bar plot showing number of genes that are differentially regulated or unchanged in NCoR1 depleted cDC1 at 6 h CpG, 6 h pIC or common CpG + pIC conditions as compared to the list of genes found to be differentially expressed in 6 h CpG, 6 h pIC and combined CpG + pIC challenged cDC1 versus unstimulated cDC1. **B** Heatmap clusters demonstrating the activation specific gene expression pattern in 6 h CpG, 6 h pIC and combined 6 h CpG + pIC challenge in control and NCoR1 KD cDC1. Important DC response genes showing signal specific responses are marked. **C** Scatter plots showing comparison of Log2 fold change in gene expression and corresponding enhancer activity at signal specific genes in CpG and pIC activated condition in NCoR1 depleted condition versus control cDC1 cells. **D** IGV browser snapshot showing NCoR1 binding enrichment in wild type cDC1 cells and H3K27ac histone mark enrichment in control and NCoR1 KD cDC1 at *Il12b*, *Il10* and *Ifnb1* gene loci. **E** Bar plots showing normalized counts (DESeq2) for selected target genes from RNA-seq data of control and NCoR1 KD cDC1s in unstimulated, CpG, pIC and CpG + pIC + IFNγ stimulation

Increased enhancer activity after NCoR1 depletion led to hypothesize that there would be an increased expression of the NCoR1 bound immune response genes in a TLR stimulation specific manner. To investigate the impact of NCoR1 KD on global gene expression changes, we analyzed the RNA-seq data performed in NCoR1 KD cDC1 before and after IFNγ, CpG, pIC, CpG + pIC + IFNγ stimulation for 6 h. We first analyzed the global changes in gene expression after NCoR1 depletion in all the stimulated conditions. We observed a total of 1385 genes upregulated in TLR9-stimulated cells, relatively similar to the number of genes upregulated in control cDC1 upon CpG challenge and 613 genes were found to be downregulated after NCoR1 KD (Supplementary Fig. 4A). On the other side, TLR3 stimulation condition showed an increase in only 865 genes upon NCoR1 depletion, which is drastically reduced in number as compared to genes upregulated in control cDC1 after TLR3 ligation (Supplementary Fig. 4A, C). Similar effects were observed in the number of genes in combined CpG + pIC + IFNγ stimulation in NCoR1 depleted cDC1 (Supplementary Fig. 4A, C). To further understand the global impact of NCoR1 depletion on gene expression patterns, we performed lrt (log likelihood ratio test) and identified a total of 5123 genes showing significant variation in expression across the stimulation conditions. Gene expression pattern analysis of this gene list showed 65% of the total genes in Cluster 1–11 having stimulation-dependent increase or decrease in gene expression (Supplementary Fig. 4D). Overall, NCoR1 KD led to an increase in the number of genes in CpG (Cluster 1, 2, 6, 7) activation, while pIC activation led to either no significant change or decrease in gene expression (Cluster 2, 3, 6, and 10). Then, we specifically looked into CpG/pIC specific or common genes, we found that NCoR1 depletion led to drastic increased expression of CpG and pIC specific as well as common CpG-pIC genes, however, in pIC stimulation condition, NCoR1 depletion led to increase in only very few pIC-specific genes. On the contrary, NCoR1 KD showed a negative effect on the number of pIC-specific antiviral genes such as *Ifnb1*, *Cxcl10*, ISGs and several other genes involved in antiviral responses (*Trim14, Trim25, Adar, Helz2*) both at 2 h and 6 h pIC stimulation, which are downregulated after NCoR1 KD in cDC1 (Fig. 5A, B, Supplementary Fig. 4E–G) [52–78]. To understand the role of NCoR1-mediated regulation of enhancer activity in regulating CpG/pIC-specific gene expression after NCoR1 KD, we correlated gene expression and acetylation activity in NCoR1 KD versus Control CpG and pIC stimulation conditions. Overall, the CpG stimulation showed a similar increase in enhancer activity and gene expression for both CpG specific, pIC specific and common CpG-pIC genes while pIC stimulation led to decrease in gene expression and enhancer activity of pIC specific genes (Fig. 5C). This observation is more robust at 2 h as compared to 6 h pIC activation condition (Supplementary Fig. 4H). NCoR1 KD CpG activated cDC1 showed increased enhancer activity and expression of major inflammatory, anti-inflammatory and antiviral genes, however, antiviral genes showed reduced enhancer activity and expression in pIC activation condition (Fig. 5D, E). We also observed decreased expression as well as enhancer activity near TSS of *Sirt1* in NCoR1 KD pIC activated cDC1 (Supplementary Fig. 4H, I)*.* Study of respiratory syncytial virus infection in SIRT1-deficient BMDC has shown increased fatty acid synthesis leading to mitochondrial dysfunction and reduced antiviral response [79]. Overall, the impact of NCoR1 KD on both gene expression and enhancer activity on TLR9 and TLR3-specific genes suggests a functional bias towards TLR9 ligation in cDC1.

### NCoR1 KD suppresses TLR3 specific signal related transcription factor (SRTFs) expression and thereby ISGs and antiviral genes

**Fig. 6:**
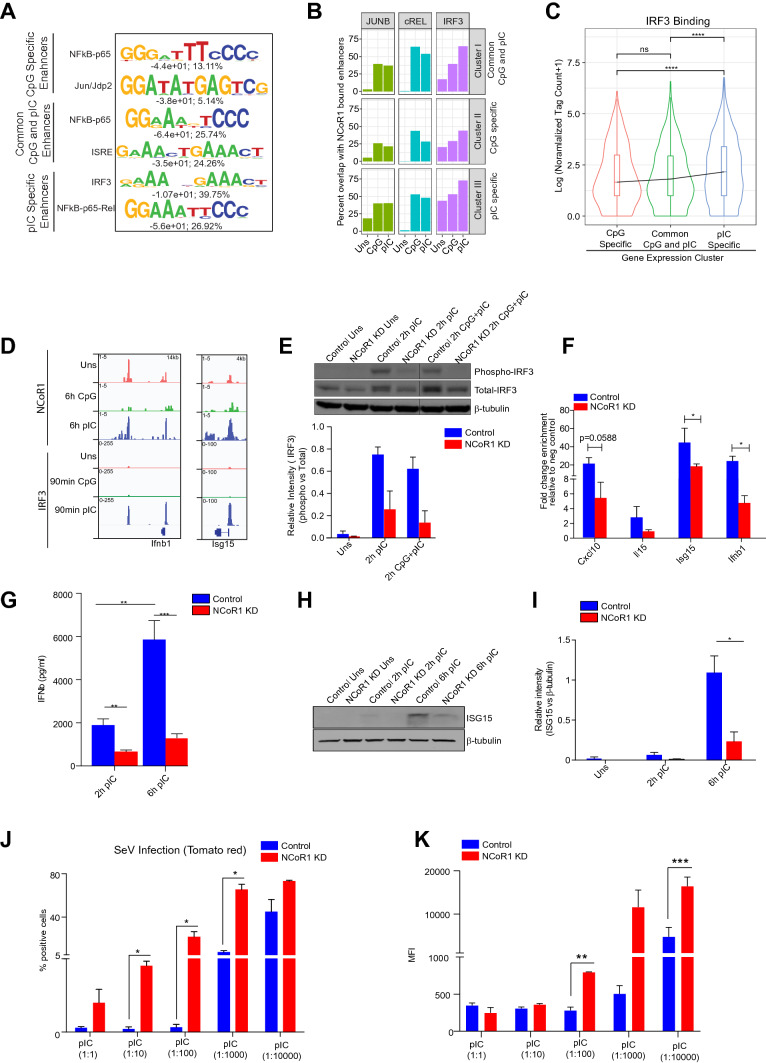
NCoR1 cistromes differentially control transcriptional regulation of pIC specific genes in cDC1. **A** Position Weight Matrix (PWM) logos for de novo enriched TF motifs on activation specific (CpG, pIC, common CpG-pIC) H3K27ac marked enhancer regions. Number below the logo represents statistical significance -log10(p-value) and percentage of target regions depicting the predicted motif. **B** Bar plot showing percent of NCoR1 bound enhancer genomic regions overlapping with JunB, cRel and IRF3 in unstimulated, CpG, and pIC activated cDC1. **C** Violin plot showing distribution of IRF3 binding intensity on CpG, pIC specific, common CpG-pIC genes. **D** IGV snapshot showing NCoR1 and IRF3 binding enrichment on *Ifnb1* and *Isg15* gene loci in unstimulated, CpG and pIC activated cDC1. **E** Western blot depicting the protein level of phosphorylated IRF3, total IRF3, and β-Tubulin housekeeping control in unstimulated, 2 h, and 6 h pIC stimulated control and NCoR1 KD cDC1. Corresponding bar plot with standard error mean shows the densitometric analysis of the western blot bands. The phospho-IRF3 band intensity was first normalized to total IRF3 followed by normalization with housekeeping control. Housekeeping gene β-Tubulin was used as protein loading control. (*n* = 3). **F** ChIP-qPCR bar plot showing the percentage enrichment of IRF3 relative to input on enhancer regions of antiviral genes (*Ifnb1, Cxcl10, Isg15, Il15*) in 2 h pIC stimulated control and NCoR1 KD cDC1. Negative control genomic regions were used to calculate the fold change in enrichment. (*n* = 3). **G** ELISA-based quantification of the secreted IFNβ cytokine in the culture supernatants of 2 h and 6 h pIC challenged NCoR1 KD and controlled cDC1 DCs (*n* = 3) **H** Western blot depicting the ISG15 and β-Tubulin housekeeping control at protein level in unstimulated, 2 h, and 6 h pIC stimulated control and NCoR1 KD cDC1 (*n* = 3). **I** Bar plot with standard error mean shows the densitometric analysis of the western blot bands of ISG15. The ISG15 band intensity was normalized to β-Tubulin (*n* = 3). **J** Bar-plot developed from flow cytometry analysis of three independent biological replicates depicting the percentage of positive SeV infected cells in control and NCoR1 KD cDC1 cells. (*n* = 3). **K** Histograms depicting MFI shifts for SeV infection from three independent biological replicates for control and NCoR1 KD cDC1 challenged with different dilutions of 6 h pIC. (*n* = 3). *p ≤ 0.05, **p ≤ 0.01 and ***p ≤ 0.001. *p* value has been calculated using the two-tailed paired student’s *t* test. Data shown in figure are combined from three independent experiments

To identify the differential impact of NCoR1 depletion on TLR9 versus TLR3 ligation mediated gene expression in cDC1, we investigated the (SRTFs) in NCoR1 depleted cells. Weighted gene co-expression network analysis (WGCNA) method is most widely used to identify the TFs-gene-regulatory network from gene expression datasets, hence we performed co-expression analysis across 5 different conditions with a total 20 samples (Supplementary Fig. 5A) [53]. We identified 13 modules represented by different colors (Supplementary Fig. 5B, C). Green and dark red modules were enriched for immune response-related pathways and the green module included the majority (28%, (*n* = 1372)) of the total genes (Supplementary Fig. 5D). We then looked into the transcription factor and co-regulators with their target genes in each of these modules. Out of the total known TFs and co-regulators, we identified 131 TFs and coregulators associated with green and darkred modules. The TFs were then ranked based on p value of association with their target genes that were found to be differentially expressed in CpG/pIC/CpG + pIC + IFNγ conditions in our dataset (see “Methods” for details) (Supplementary Fig. 5E and Table S4). We also identified known interactions of TFs and their target genes from the StringDB database for green and darkred module. In the TF-gene-regulatory-networks, we found several of the known regulators of TLR9/TLR3 response genes enriched, such as Irf7, Rel, RelB, Stat1, Stat2, Stat3 and Irf9 [23] (Supplementary Fig. 5E). We found that several of the highly expressed pIC-specific SRTFs were downregulated including RelB and cRel after pIC challenge in NCoR1 KD condition (Supplementary Fig. 5F). Furthermore, the de novo TF motif enrichment analysis on the enhancers overlapping with differential NCoR1-binding sites associated with TLR9/TLR3-specific genes revealed predominance of NFkB-p65 (RelA) and JunB motifs on enhancers of TLR9-specific genes. On the other hand, common TLR9 and TLR3 and only TLR3-specific genes associated enhancers were enriched for both NFkB and ISRE/IRF3 motifs (Fig. 6A and Supplementary Fig. 5G). To experimentally validate the predicted TF bindings, we first mapped JunB, cRel and Irf3 using ChIP-seq data generated in the same cell line in unstimulated, CpG or pIC challenged conditions on NCoR1 bound regions. We observed an increased JunB, cRel-binding enrichment in both CpG and pIC stimulation (Supplementary Fig. 6A). Overlap of NCoR1 with JunB and cRel peaks showed an overlap of 25–50%. IRF3 bindings were found to be increased after pIC activation compared to CpG and showed 50–75% overlap with NCoR1 peaks in all the NCoR1-binding clusters (Fig. 6B, Supplementary Fig. 6B). Furthermore, we mapped TF and H3K27ac ChIP-seq binding data from primary bone marrow derived dendritic cells (BMDCs) upon LPS stimulation to confirm the binding intensity of these TFs and H3K27ac level on different categories of stimulation dependent NCoR1 binding. We found a similar binding intensity profile for these TFs as well as H3K27ac across all the NCoR1 binding clusters, as observed in the cDC1 cell line (Supplementary Fig. 6C). We then compared the cRel and IRF3 binding on NCoR1-binding sites associated with CpG, pIC specific and common CpG-pIC genes. cRel binding showed significantly high enrichment in CpG compared to pIC on CpG specific as well as common CpG-pIC genes while pIC-specific genes showed similar enrichment in CpG and pIC condition (Supplementary Fig. 6D). On the other hand, IRF3 binding was found to be significantly more enriched in pIC activation condition on all gene categories (Fig. 6C, D, Supplementary Fig. 6D). Among the TFs based on motif prediction and TFs ChIP-seq enrichment on NCoR1-binding sites we looked into expression of IRF3 at protein level and its binding at key enhancer region of antiviral gene loci as IRF3 is one of the major TLR3-specific TF controlling the antiviral response genes and ISGs. We found a decreased trend in pIRF3 expression in NCoR1 depleted pIC challenged cDC1 at both 2 h pIC and 2 h CpG + pIC stimulation (Fig. 6E). Furthermore, ChIP-qPCR analysis of IRF3 binding on enhancers of important antiviral genes in control and NCoR1 KD cDC1 showed decreased enrichment in NCoR1 KD compared to control cDC1 (Fig. 6F). To confirm the decrease in transcript expression of IFNb1 and ISGs due to decreased IRF3 phosphorylation at protein level, we performed ELISA and western for IFNb1 and ISG15, respectively. NCoR1 depleted 2 h and 6 h pIC activation conditions showed decreased IFNb1 and ISG15 expression at protein level (Fig. 6G–I).

### TLR3 activated NCoR1 KD cDC1 leads to decreased antiviral response and T cell cytotoxicity

The functional impact of decreased antiviral gene expression in NCoR1 KD cells was validated using the Sendai virus (SeV) infection model. cDC1 cells were preincubated with pIC at different concentrations and the antiviral effect generated was observed through percent positive cells infected with tomato red tagged SeV. NCoR1 KD cDC1 indeed showed decreased trend in antiviral response at absolute concentration and significantly decreased antiviral response till 1:1000 dilution of pIC stimulation as indicated by the percent positive cell and Median fluorescence intensity (MFI) when incubated with SeV compared to control cDC1 in flow cytometry (Fig. 6J, K and Supplementary Fig. 7A, B). Since cDC1 is well known for CD8^+^ T-cell-mediated immune responses, we tried to identify the potential of these pIC-activated NCoR1 KD DCs to modulate cytotoxic activity of CD8^+^ T-cells. No significant change was observed in the proliferation of OT-I CD8 ^+^ T-cells co-cultured with NCoR1 KD cDC1 as compared to control DCs activated with pIC at different dilutions (Supplementary Fig. 7C, D). For flow cytometry analysis, we used a uniform gating strategy across all the samples (Supplementary Fig. 7E). Furthermore, we also checked intracellular expression of Perforin, Granzyme-B and IFN-γ to understand the cytotoxic potential of co-cultured OT-I CD8^+^ T-cells. First, effector CD8^+^ T-cells were gated based on CD3^+^CD8^+^CD44^+^ markers and percent positive and MFI were estimated for cells expressing Perforin, Granzyme B and IFN-γ. We observed significant decrease in cytotoxicity potential in NCoR1 KD cDC1 compared to control activated with pIC at dilution 1:10 (Supplementary Fig. 7F–H). Back gating strategies were followed uniformly across all samples (Supplementary Fig. 7I**)**.

## Discussion

Immune responses such as inflammatory, anti-inflammatory and antiviral under TLR9 or TLR3 stimulation in DCs are required to clear the pathogen. Role of transcription factor binding on the cis-regulatory element upon TLR activation e.g. NFkB, IRFs or STATs has been elucidated as an important regulator of immune response genes in DCs [80]. However, binding of these transcription factors are determined majorly by the accessibility of the chromatin in the regulatory regions of their target genes [81, 82]. Co-repressors protein complexes that include NCoR1 play an important role in regulating spatio-temporal activity of enhancers controlling the binding of TFs thereby maintaining tight regulation of expression of immune response genes [83]. To understand the regulation of TLR specific enhancer activity regulated through NCoR1, we mapped enhancers under TLR9 and TLR3 activation using H3K27ac ChIP-seq data in control and NCoR1 depleted condition and NCoR1 binding using NCoR1 ChIP-seq data. Moreover, as TLR9 and TLR3 stimulation leads to activation of transcription factors under two different signaling pathways, we investigated the effect of TLR9 activation on TLR3 response and vice versa through mapping of enhancer activity and gene expression in combined TLR9 and TLR3 stimulation.

Gene expression data revealed genes specific to TLR9, TLR3 and common between TLR9 and TLR3 and also synergistic and antagonist activity of either TLR in combined stimulation. IFNγ stimulation was used as a host factor, however, since IFNγ either in an individual or combined stimulation with TLR9 + TLR3 did not show any major effect on TLR response genes, hence we performed H3K27ac ChIP-seq in only TLR9 + TLR3 stimulation. Similar to gene expression, mapping of enhancer activity using H3K27ac ChIP-seq data in TLR activated cDC1 revealed temporal activity of enhancers specific to TLR9 and TLR3. The enhancer activity showed an early increase in activity at 2 h that further attenuated at 6 h in TLR3 activation whereas TLR9 showed delayed activity at 6 h of stimulation. Moreover, both enhancer activity and gene expression showed the dominant role of TLR9 activation in combined TLR9 and TLR3 in cDC1 DCs. As enhancers are mostly present on far distal regions to TSS, many of the TLR9/TLR3 specific genes were found to be associated with the SE regions. TLR3 and TLR9-specific super enhancers’ activity were also observed at early 2 h and late 6 h stimulation, respectively. Further synergistic and antagonist effects of TLR9/TLR3 on enhancer activity in combined stimulation was observed for genes that showed similar phenomenon at transcriptional level.

TLR9/TLR3 activation-dependent NCoR1 binding revealed that majority of the NCoR1-binding sites falls in (~ 85%) far distal regions to TSS and ~ 50% of NCoR1-binding sites does not show any change in binding enrichment at 6 h of TLR3/TLR9 activation, while only 4% shows decrease in binding enrichment after TLR9/TLR3 activation. TLR3/TLR9-specific NCoR1-binding were found to be drastically increased upon TLR9 activation on majority of the genomic regions, however genomic regions having TLR3 specific increase in binding were found to be slightly enriched in unstimulated condition again indicating that repression of enhancers activity in cDC1 is biased towards TLR9 activation. TLR3/TLR9 stimulation specific strong correlation of enhancer activity with NCoR1 binding led us to hypothesize the regulation of enhancer activity through NCoR1 and further increase in enhancer activity upon TLR9/TLR3 stimulation in time dependent manner after NCoR1 depletion substantiated our finding that NCoR1 acts a strong repressor of TLR9/TLR3 specific enhancers.

Gene expression analysis of NCoR1 depleted DCs revealed that strong repressive activity of NCoR1 on majority of the TLR9/TLR3-specific genes upon TLR9 stimulation exemplified by significant increase in expression of inflammatory (*Il12b, Il1b, Il6*) tolerogenic (*Il10, Socs3*) and antiviral genes (*Ifnb1, Cxcl10, Il15*). However, on the other hand, the repressive activity of NCoR1 on the TLR9/TLR3-specific genes could not be observed in case of TLR3 stimulation as there is significant decrease in expression of TLR3-specific genes. And similarly, the enhancer activity of TLR3-specific genes were found to be decreased in NCoR1 depleted TLR3 stimulated DCs.

Furthermore, our gene co-expression analysis along with TFs motif enrichment analysis on enhancers and NCoR1-binding sites overlap with TFs ChIP-seq data suggested that NFkB family TFs under TLR9 activation is an important regulator of TLR9/TLR3-specific genes for increase in gene expression after NCoR1 depletion, while on the other hand, IRF3 is important transcriptional regulator of TLR3 specific NCoR1 bound site and its target genes under TLR3 activation. Decreased protein level expression of IRF3 and its binding on key enhancer regions of an important antiviral gene in NCoR1 depleted DCs further confirmed the differential and opposite role of NCoR1 in TLR3 activation compared to TLR9. Moreover, decreased antiviral response and cytotoxic potential of CD8^+^ T-cells in NCoR1 depleted TLR3 activated DCs also further substantiated its differential and opposite role in cDC1 DCs. Studies in macrophage have shown that NCoR1 depletion leads to increased fatty acid oxidation through derepression of LXR and also increased fatty acid oxidation pathway is known to have a role in limiting virus infection in DCs through *Sirt1* [79, 84]. Decreased *Sirt1* expression along with enriched fatty acid metabolism pathway and increased *Pparg* expression in NCoR1 KD TLR3 activated cDC1 hints towards increased fatty oxidation might be also an important pathway leading to decreased antiviral response (Supplementary Fig. 4B and H, I).

In conclusion, overall our genomic, transcriptomic and epigenomic study in cDC1 DCs suggests that NCoR1-mediated repression of immune response is skewed or biased towards TLR9 compared to TLR3.

### Supplementary Information

Below is the link to the electronic supplementary material.

Supplementary file1 (PDF 8726 KB)

Supplementary file2 (PDF 1877 KB)

Supplementary file3 (PDF 5080 KB)

Supplementary file4 (PDF 1551 KB)

Supplementary file5 (PDF 2373 KB)

Supplementary file6 (PDF 33793 KB)

Supplementary file7 (PDF 1276 KB)

Table S1: Excel file having list of genes specific to CpG, pIC, common between CpG and pIC, synergistic and antagonist genes in combined CpG+pIC identified based on differential expression analysis of Control CpG and pIC stimulated cDC1 DCs. (XLSX 57 KB)

Table S2: Excel file having list of H3K27ac peaks showing enhancer activity specific to CpG, pIC, common between CpG and pIC and down (decreased activity after stimulation). (XLSX 275 KB)

Table S3: Excel file having NCoR1 peaks cluster with its annotation. (XLSX 3630 KB)

Table S4: Excel file having list of Transcription factor identified in green and darkred module with its significance of association with target genes differentially expressed in NCoR1 KD CpG, pIC and CpG+pIC+IFNγ stimulated DCs compared to respective control. (XLSX 37 KB)

## Data Availability

All the high throughput sequencing data are available at EMBL-EBI ArrayExpress database under the accession number E-MTAB-10989, E-MTAB-10991 and E-MTAB-10992. Few other datasets used in this study have been deposited previously to NCBI Gene Expression Omnibus database and available under the accession number and GSE110423. The R code used in this study has been deposited to GitHub https://github.com/sraghav-lab/NCoR1-TLR9-TLR3-Project.
