# Supplementary material for: Epigenomics of conventional type-I dendritic cells depicted preferential control of TLR9 versus TLR3 response by NCoR1 through differential IRF3 activation
Source: Cell Mol Life Sci. 2022 Jul 18;79(8):429. doi: 10.1007/s00018-022-04424-w (PMC9293861; doi:10.1007/s00018-022-04424-w)
Supplement: Supplementary file 1 — Supplementary file1 (PDF 8726 KB) [file 18_2022_4424_MOESM1_ESM.pdf]

Supplementary Figure 1

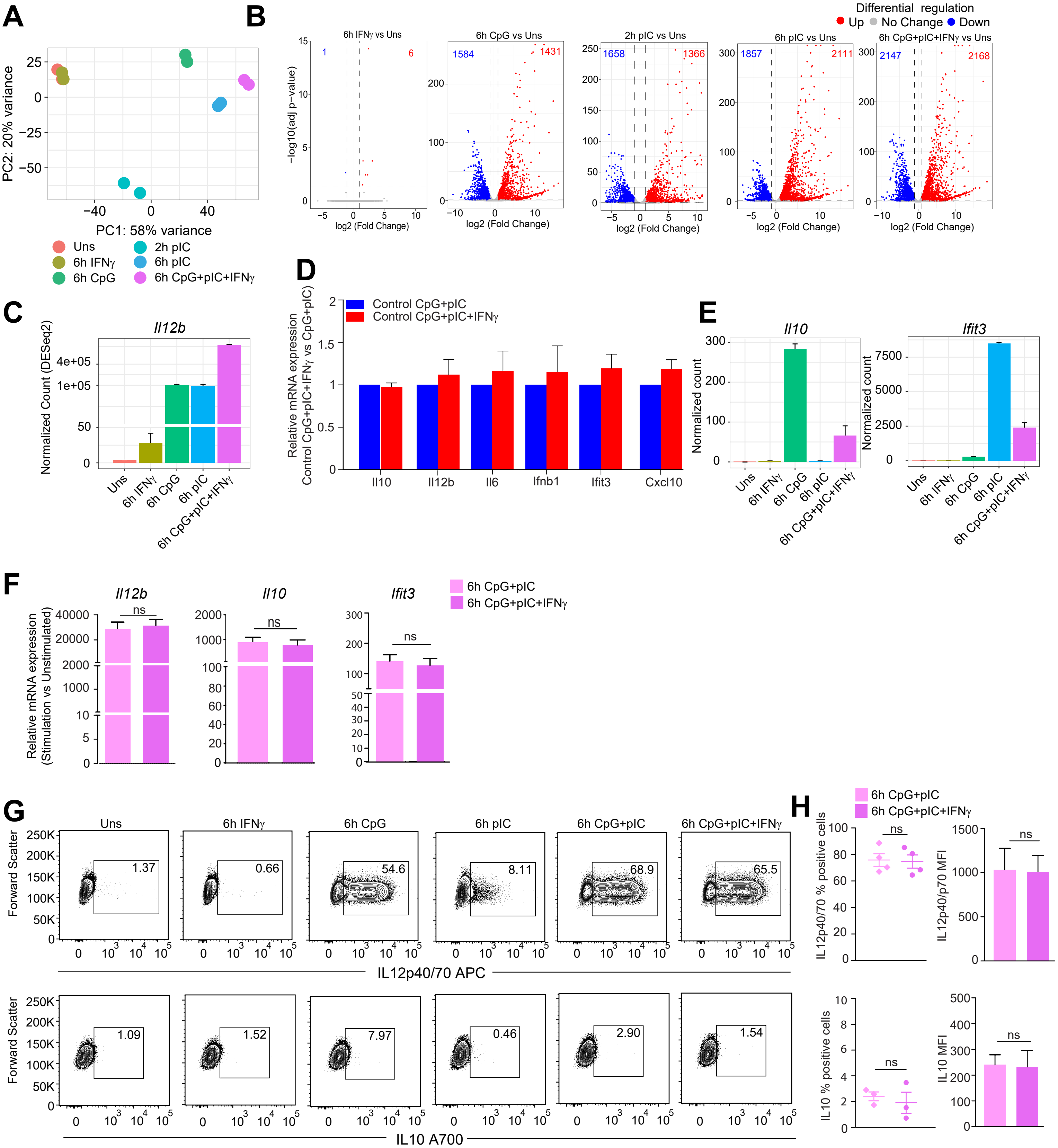

Figure S1. RNA-seq analysis of unstimulated and TLR activated cDC1

(A). PCA plot showing percent of variance explained by principal component 1 and 2 among unstimulated, IFNγ, CpG, pIC and CpG+pIC+IFNγ stimulation conditions in cDC1. **(B).** Volcano plot showing total number of differentially upregulated and downregulated genes at IFNγ, CpG, pIC and CpG+pIC+IFNγ activated DCs compared to unstimulated. **(C).** Bar plot showing DESeq2 normalized count of *Il12b* in TLR/IFNγ activated cDC1. **(D).** RT-qPCR showing relative mRNA expression of TLR response genes in 6h CpG+pIC+IFNγ vs 6h CpG+pIC stimulation condition in control cDC1 (n=3). **(E).** Bar plot showing DESeq2 normalized count of exemplary DC response genes showing antagonistic expression behavior after combined 6h CpG+pIC+IFNγ stimulation. **(F).** RT-qPCR showing relative mRNA expression of exemplary TLR response genes showing no significant difference between 6h CpG+pIC and CpG+pIC+IFNγ stimulation conditions in control cDC1 (n=6). **(G).** Contour plot from flow cytometry analysis depicting the intracellular cytokine expression of IL-12p40/70, IL-10, in control cDC1 before and after 6h TLR/IFNγ stimulation. **(H).** Scatter dot plot and bar-plot representing percent positive cells and MFI respectively for IL-12b (n=4) and IL-10 (n=3).
