# Supplementary material for: Epigenomics of conventional type-I dendritic cells depicted preferential control of TLR9 versus TLR3 response by NCoR1 through differential IRF3 activation
Source: Cell Mol Life Sci. 2022 Jul 18;79(8):429. doi: 10.1007/s00018-022-04424-w (PMC9293861; doi:10.1007/s00018-022-04424-w)
Supplement: Supplementary file 2 — Supplementary file2 (PDF 1877 KB) [file 18_2022_4424_MOESM2_ESM.pdf]

Supplementary Figure 2

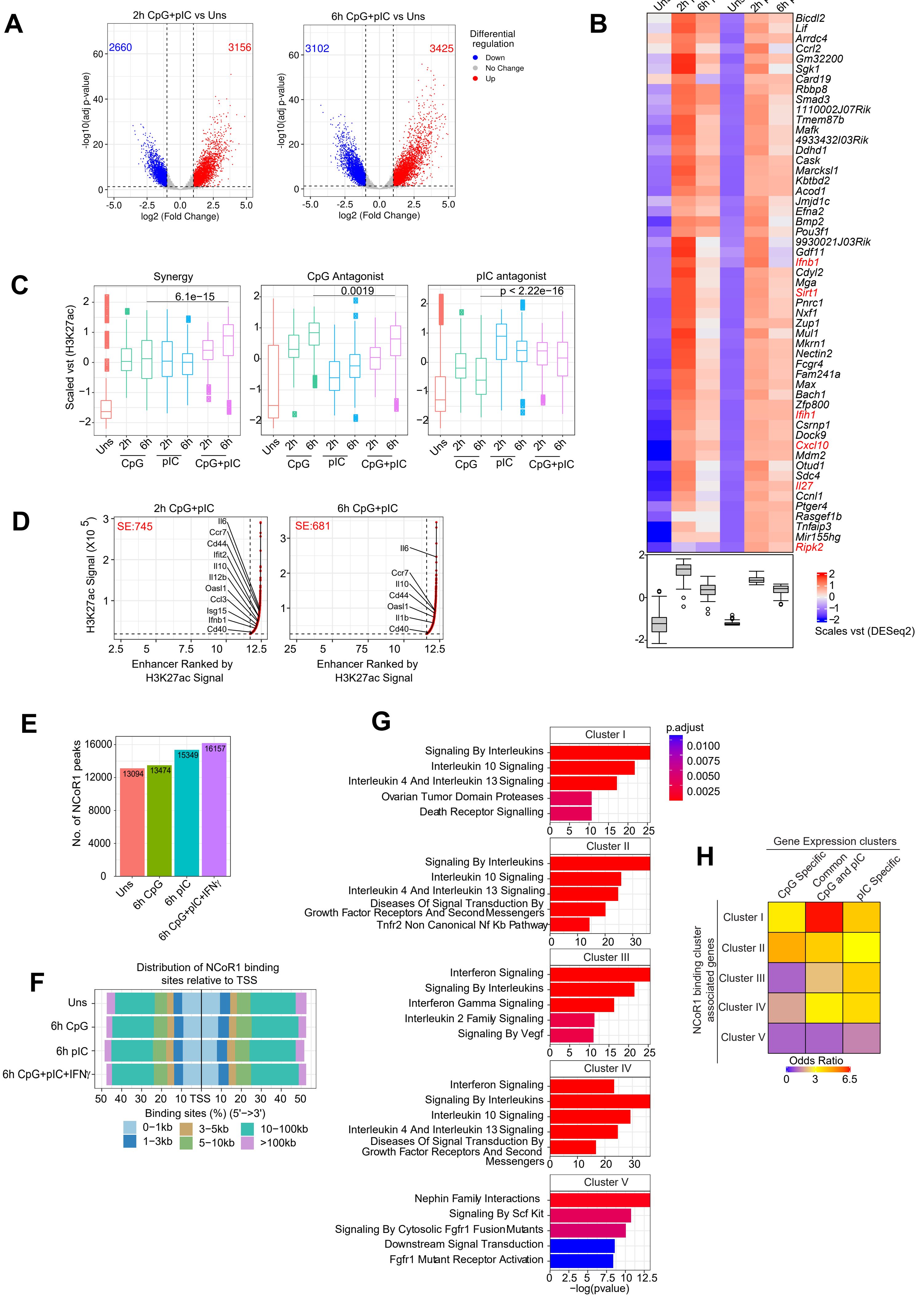

Figure S2. H3K27ac enrichment density and NCoR1 binding analysis.

(A). Volcano plot showing total number of differentially acetylated regions at 2h and 6h CpG+pIC activated DCs compared to unstimulated. **(B).** Heatmap showing scaled vst of H3K27ac enrichement from ChIP-seq and gene expression from RNA-seq data of pIC specific genes in unstimulated, 2h and 6h stimulated condition **(C).** Boxplot showing distribution of scaled vst H3K27ac enrichmnet on syngergistic, CpG and pIC antagoized genes. **(D).** Scatter plots showing the Super Enhancers (SE) identified at 2hr, and 6hr CpG+pIC stimulated cDC1. The CpG/pIC specific genes annotated to the SE regions have been marked for each condition. **(E).** Bar plot showing total number of NCoR1 binding sites identified in unstimulated, 6h CpG, pIC and CpG+pIC+IFNγ stimulation conditions in cDC1. **(F).** Percent stacked bar plot showing percent of distribution of NCoR1 binding sites distance relative to TSS in unstimulated, 6h CpG, pIC and CpG+pIC+IFNγ stimulation conditions in cDC1. **(G).** Bar plot showing significantly enriched Reactome term from MSigDB for each NCoR1 binding cluster. **(H).** Heatmap showing odds ratio of overlap of genes associated to each NCoR1 binding cluster and genes classified based on expression as CpG, pIC specific and common CpG-pIC.
