# Supplementary material for: Epigenomics of conventional type-I dendritic cells depicted preferential control of TLR9 versus TLR3 response by NCoR1 through differential IRF3 activation
Source: Cell Mol Life Sci. 2022 Jul 18;79(8):429. doi: 10.1007/s00018-022-04424-w (PMC9293861; doi:10.1007/s00018-022-04424-w)
Supplement: Supplementary file 3 — Supplementary file3 (PDF 5080 KB) [file 18_2022_4424_MOESM3_ESM.pdf]

Supplementary Figure 3

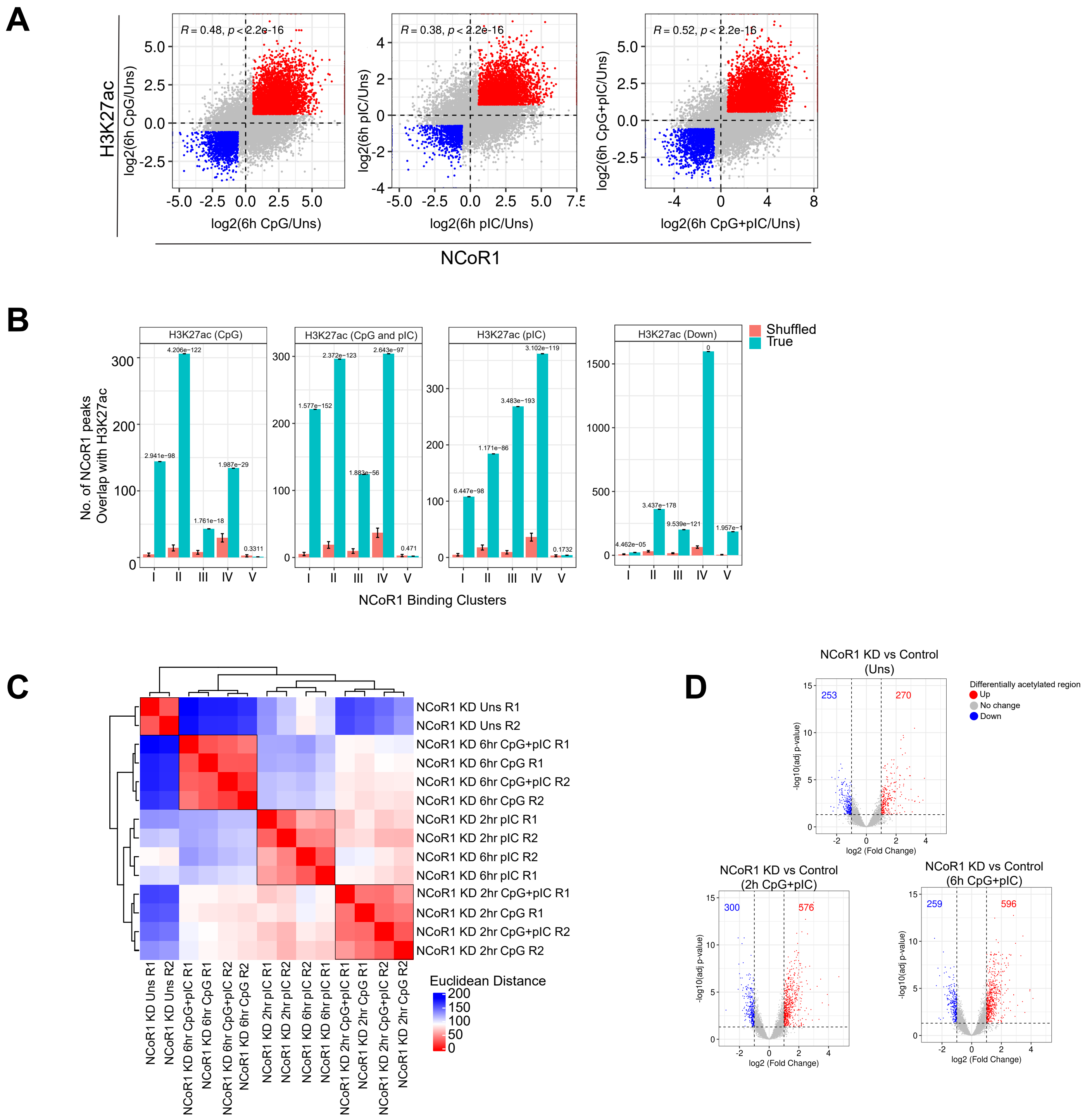

Figure S3. Overlap of NCoR1 with H3K27ac marked enhancer and effect of enhancer activity in NCoR1 depleted cDC1.

(A). Scatter plot showing correlation of ratio of normalized tag count of NCoR1 binding enrichment in the CpG, pIC and CpG+pIC stimulation compared to unstimulated versus H3K27ac ChIP enrichment in the ± 500bp to NCoR1 peak center in CpG, pIC and CpG+pIC stimulation condition compared to unstimulated. **(B).** Bar plot showing number of overlapping genomic regions of NCoR1 binding cluster with enhancer clusters defined based on H3K27ac ChIP-seq data shown in Figure 2C. Significance of overlap was calculated using OLOGRAM program. **(C).** Heatmap showing hierarchical clustering of NCoR1 KD H3K27ac ChIP-seq samples in unstimulated, 2h, and 6h (CpG, pIC and CpG+pIC) stimulated condition based on euclidean distance. **(D).** Volcano plot showing total number of differentially acetylated genomic regions in unstimulated, 2h and 6h CpG+pIC stimulated NCoR1 KD cDC1 compared to respective control samples.
