# Supplementary material for: Epigenomics of conventional type-I dendritic cells depicted preferential control of TLR9 versus TLR3 response by NCoR1 through differential IRF3 activation
Source: Cell Mol Life Sci. 2022 Jul 18;79(8):429. doi: 10.1007/s00018-022-04424-w (PMC9293861; doi:10.1007/s00018-022-04424-w)
Supplement: Supplementary file 4 — Supplementary file4 (PDF 1551 KB) [file 18_2022_4424_MOESM4_ESM.pdf]

Supplementary Figure 4

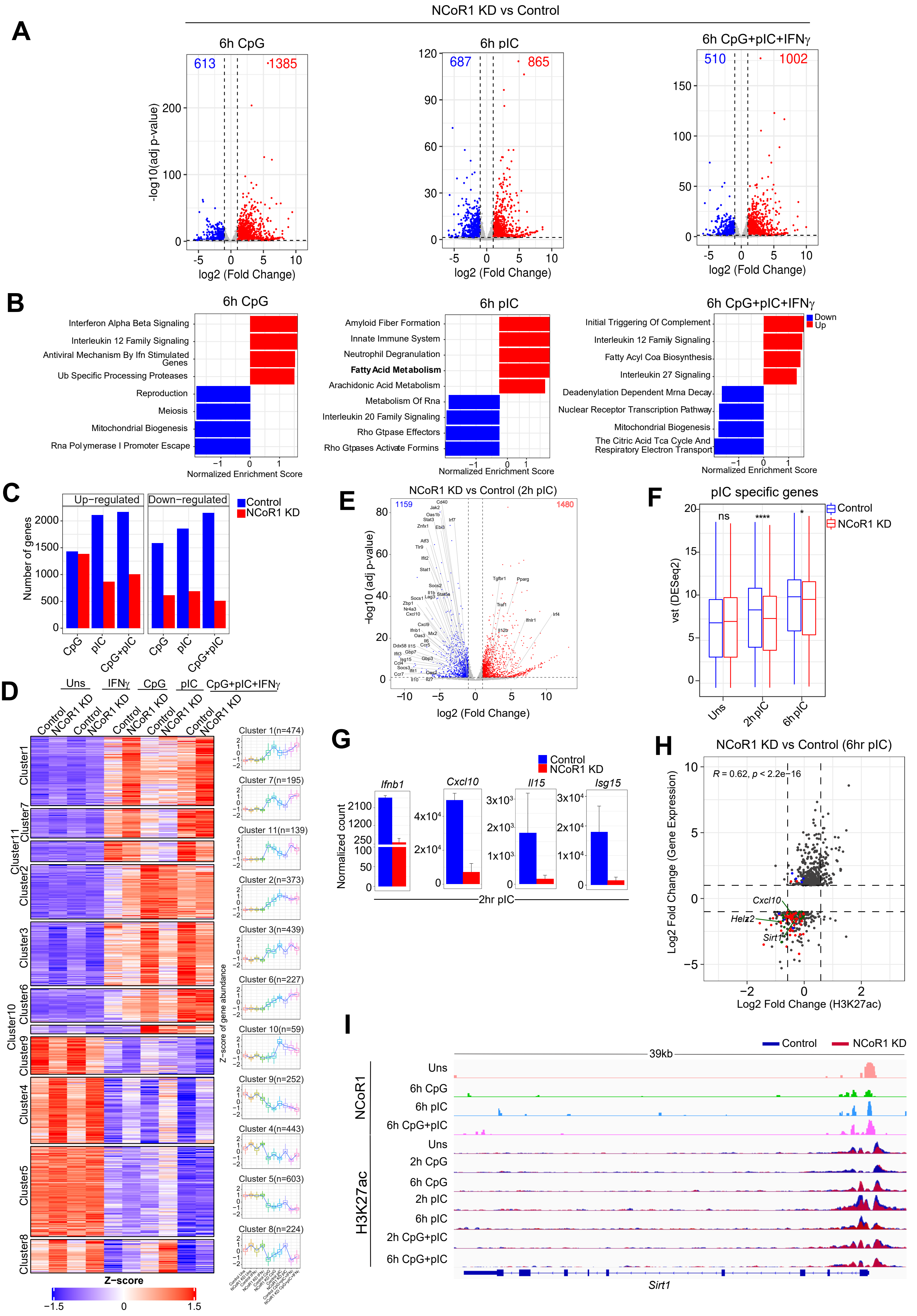

Figure S4. RNA-seq analysis of unstimulated and TLR activated NCoR1 KD cDC1

(A). Volcano plot showing total differentially upregulated and downregulated genes in NCoR1 KD 6h CpG, 6h pIC and 6h CpG+pIC+IFNγ compared to respective stimulated control activation condition. **(B).** Bar plot showing normalized enrichment score of enriched Reactome term for total DEGs in NCoR1 KD 6h CpG, 6h pIC and 6h CpG+pIC+IFNγ compared to respective stimulated control activation condtion. **(C).** Bar plot showing comparison of number of differentially up and down regulated gene before and after NCoR1 KD at 6h CpG, 6h pIC and 6h CpG+pIC+IFNγ stimulated condition. **(D).** Heatmap showing cluster (1-11) that are stimulation dependent increase or decrease in expression in Control or NCoR1 KD cDC1. **(E).** Volcano plot showing total differentially upregulated and downregulated genes in NCoR1 KD 2h pIC, compared to Control 2h pIC. Major immune response genes have been marked in the plot. **(F).** Boxplot showing distribution of vst (DESeq2) expression value for pIC specific genes at 0h, 2h pIC and 6h pIC in Control and NCoR1 KD cDC1. Significance of differrence between mean were calculated based on Wilcoxon test. **(G).** Bar plot showing normalized count for TLR3 resposne genes in 2h pIC activation condition in cotnrol and NCoR1 KD cDC1. **(H)** Scatter plots showing comparison of Log2 fold change in gene expression and corresponding enhancer activity at 6h pIC activated condition in NCoR1 depleted condition versus control cDC1 cells. **(I).** IGV browser snapshot showing NCoR1 binding enrichment in wild type cDC1 cells and H3K27ac histone mark enrichment in control and NCoR1 KD cDC1 at Sirt1 gene loci.
