# Supplementary material for: Epigenomics of conventional type-I dendritic cells depicted preferential control of TLR9 versus TLR3 response by NCoR1 through differential IRF3 activation
Source: Cell Mol Life Sci. 2022 Jul 18;79(8):429. doi: 10.1007/s00018-022-04424-w (PMC9293861; doi:10.1007/s00018-022-04424-w)
Supplement: Supplementary file 5 — Supplementary file5 (PDF 2373 KB) [file 18_2022_4424_MOESM5_ESM.pdf]

Supplementary Figure 5

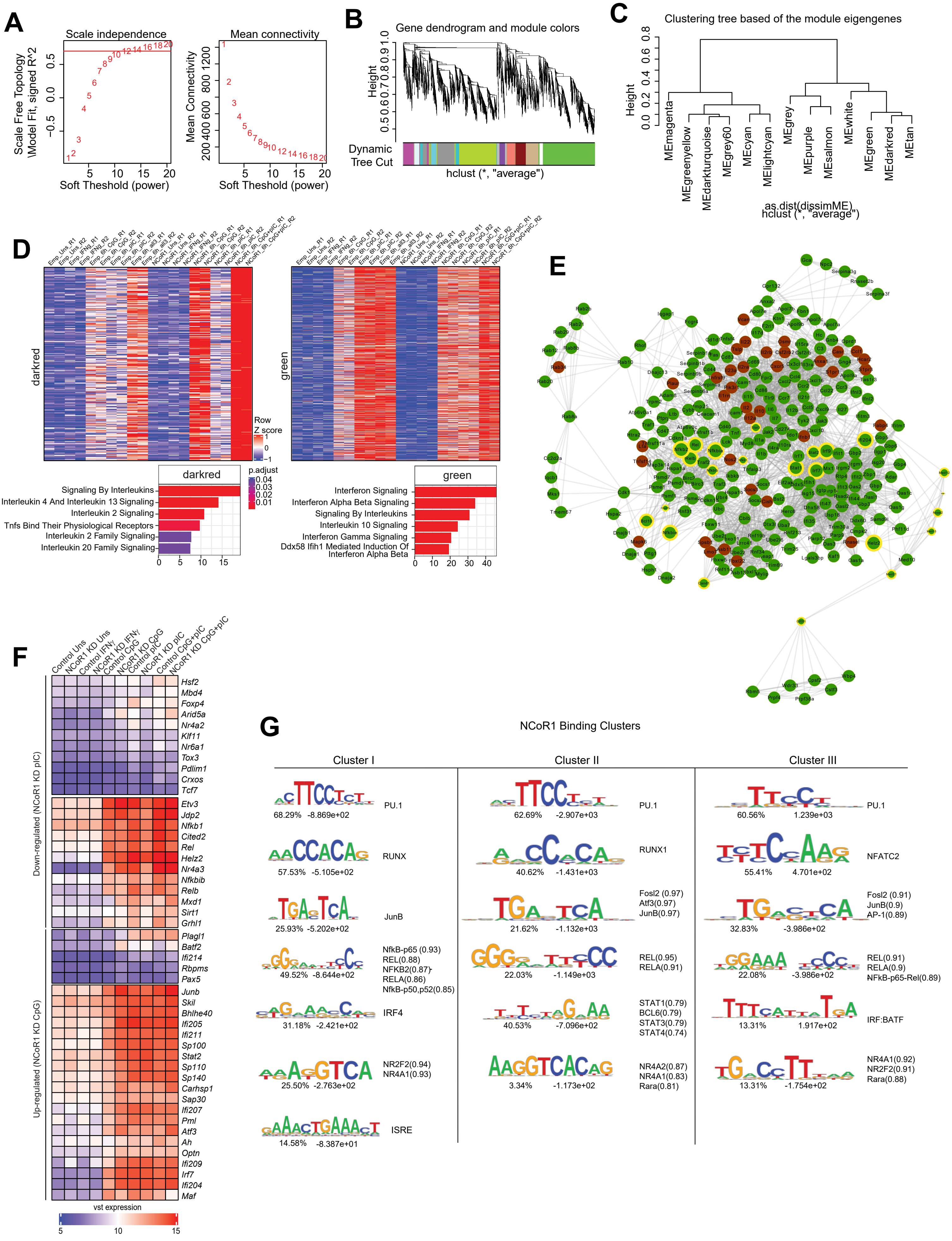

Figure S5. Gene co-expression network analysis and Motif enrichment analysis on differential NCoR1 binding sites

(A). Analysis of scale-free fit index and mean connectivity of various soft thresholding power. **(B).** Dendrogram of all differentially expressed genes clustered, based on a dissimilarity measure (1-TOM). **(C).** Tree dendrogram showing module network constructed by clustering module eigengene distances. **(D).** Heatmap showing pattern of gene expression from darkred and green module and its associated enriched pathway. **(E).** Gene-gene network showing interaction of transcription factor and its target gene identified from WGCNA and also interaction known from StringDB database. **(F).** Heatmap showing expression of transcription factor found to be enriched darkred and green module and ranked based on differential expression of its target genes in NCoR1 KD CpG, pIC and CpG+pIC stimulated condition compared to control. **(G).** TFs motif identified after *De Novo* motif enrichment analysis on genomic regions of different NCoR1 binding clusters. Number shown below the motif logo represents percent of the target regions showing a particular motif and -log10(p-value) represents statistical significance calculated in comparison to back ground sequence using HOMER.
