# Supplementary material for: Epigenomics of conventional type-I dendritic cells depicted preferential control of TLR9 versus TLR3 response by NCoR1 through differential IRF3 activation
Source: Cell Mol Life Sci. 2022 Jul 18;79(8):429. doi: 10.1007/s00018-022-04424-w (PMC9293861; doi:10.1007/s00018-022-04424-w)
Supplement: Supplementary file 6 — Supplementary file6 (PDF 33793 KB) [file 18_2022_4424_MOESM6_ESM.pdf]

Supplementary Figure 6

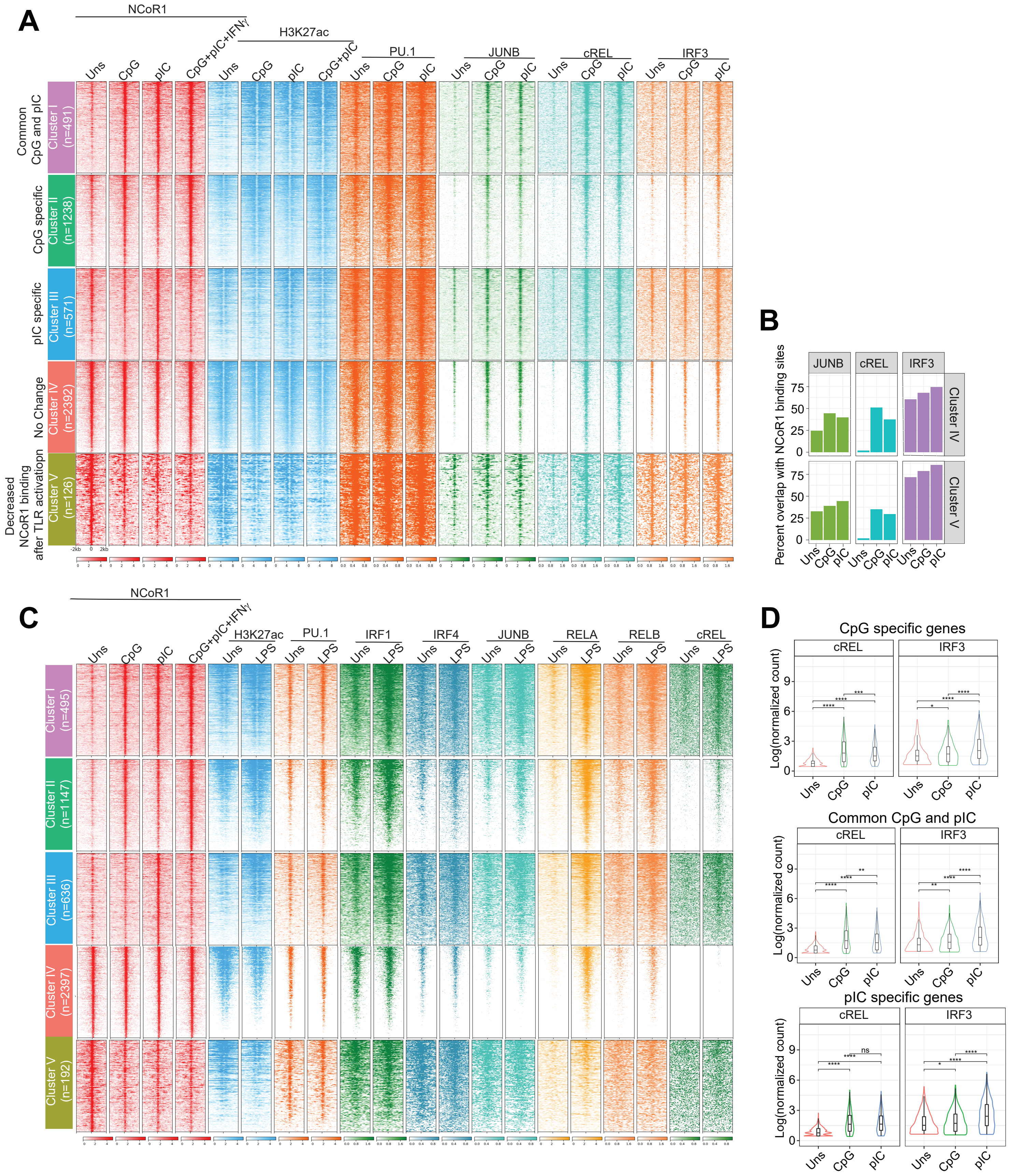

Figure S6. Analysis of publicaly available Transcription factor (TFs) ChIP-seq datasets

(A). Heatmap showing enrichment of NCoR1 and H3K27ac in Uns, CpG, pIC and CpG+pIC at ±2kb to NCoR1 peak center of genomic regions overlapping with H3K27ac peak and are associated with CpG/pIC specific genes. Similarly enrichment of TFs in cDC1 line in Uns, CpG, pIC were plotted. **(B)** Bar plot showing percent of genomic regions in each Cluster III and IV of NCoR1 binding cluster overlapping with JUNB, cREL and IRF3 in unstimulated, CpG, and pIC activated cDC1. **(C).** Heatmap showing enrichment of different TFs in BMDCs in Uns and 2h LPS stimulation condition at ±2kb to NCoR1 peak center of genomic regions overlapping with H3K27ac peaks in different NCoR1 binding clusters. **(D).** Violin plot showing distribution of normalized tag count of cRel and IRF3 at CpG, pIC specific and common CpG and pIC genes in Uns, CpG and pIC stimulation condition. Statistical significance was calculated using the Wilcoxon test.
