# Supplementary material for: Epigenomics of conventional type-I dendritic cells depicted preferential control of TLR9 versus TLR3 response by NCoR1 through differential IRF3 activation
Source: Cell Mol Life Sci. 2022 Jul 18;79(8):429. doi: 10.1007/s00018-022-04424-w (PMC9293861; doi:10.1007/s00018-022-04424-w)
Supplement: Supplementary file 7 — Supplementary file7 (PDF 1276 KB) [file 18_2022_4424_MOESM7_ESM.pdf]

Supplementary Figure 7

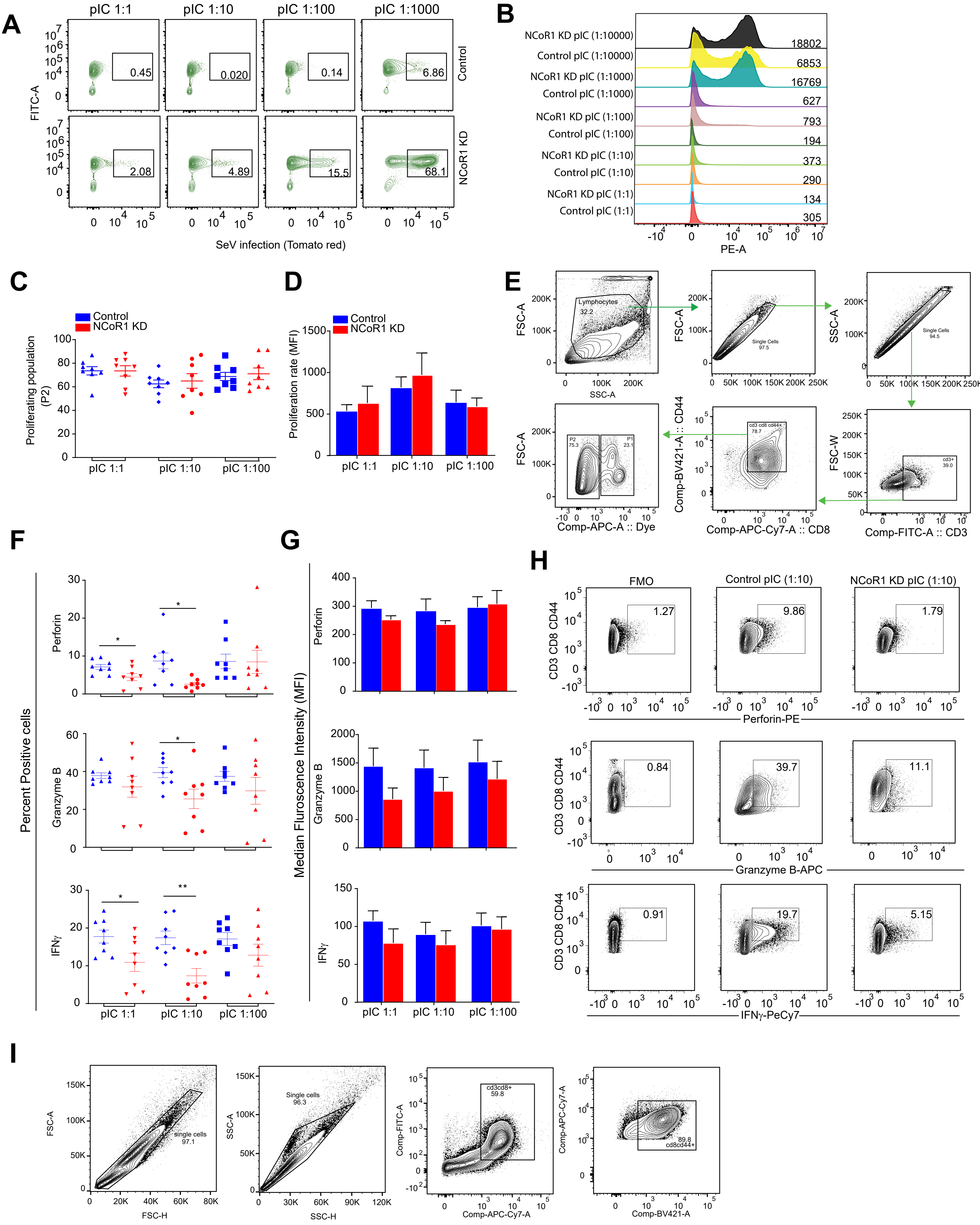

Figure S7. SeV infection in control and NCoR1 KD pIC stimulated cDC1.

(A). Representative flow cytometry contour plot depicting the SeV infected percentage positive cells in control and NCoR1 KD cDC1 upon 6h stimulation with different dilutions of pIC. Serial dilutions starting from 5µg/ml were used for the stimulation (n=3). **(B).** Representative Median Fluorescence Intensity (MFI) histogram plots showing the shift in the fluorescent intensity depicting the quantitation of SeV infection in different dilutions of 6h pIC challenged control and NCoR1 KD cDC1 (n=3). **(C).** Scatter dot plots showing proliferation of OT-I T-cells co-cultured with control and NCoR1 KD cDC1s pulsed with OVA 257-264 peptide overnight followed by different dilutions of pIC challenge for 2h. **(D).** Bar-plot demonstrating proliferation rate of OT-I T-cells co-cultured with control and NCoR1 KD DC1s pulsed with OVA peptide overnight followed by different dilutions of pIC challenge for 2h. (n=4).

(E) Back gating strategy used for flow cytometry analysis of co-cultured OT-I T-cells for checking proliferation.

(F) Flow cytometry analysis showing the scatter dot plots representing co-cultured OT-I T-cells showing cytolytic Perforin, Granzyme B, and IFN-γ in control and NCoR1 KD DCs in pIC stimulated condition. (n=4)

(G) Bar plot demonstrating MFI of Perforin, Granzyme B, and IFN-γ of co-cultured OT-1 T cells in control versus NCoR1 KD cDC1co-cultured OT-I T-cells pulsed with OVA and stimulated with difference dilutions of pIC for 2h.

(H) Flow cytometry analysis showing the contour plots representing co-cultured OT-I T-cells showing cytolytic Perforin, Granzyme B, and IFN-γ in control and NCoR1 KD DCs in pIC stimulated condition.

(I) Back gating strategy used for flow cytometry analysis of co-cultured OT-I T-cells for checking cytotoxic activity.
